# Safety and efficacy of human embryonic stem cell-derived astrocytes following intrathecal transplantation in SOD1^G93A^ and NSG animal models

**DOI:** 10.1186/s13287-018-0890-5

**Published:** 2018-06-06

**Authors:** Michal Izrael, Shalom Guy Slutsky, Tamar Admoni, Louisa Cohen, Avital Granit, Arik Hasson, Joseph Itskovitz-Eldor, Lena Krush Paker, Graciela Kuperstein, Neta Lavon, Shiran Yehezkel Ionescu, Leonardo Javier Solmesky, Rachel Zaguri, Alina Zhuravlev, Ella Volman, Judith Chebath, Michel Revel

**Affiliations:** 1 Neurodegenerative Diseases Department at Kadimastem Ltd, Pinchas Sapir 7, Weizmann Science Park, Nes-Ziona, Israel; 2 Department of Molecular Genetics, Weizmann Institute of Science, 76100 Rehovot, Israel

**Keywords:** Amyotrophic lateral sclerosis, Astrocytes, Human embryonic stem cells, Superoxide dismutase 1

## Abstract

**Background:**

Amyotrophic lateral sclerosis (ALS) is a motor neuron (MN) disease characterized by the loss of MNs in the central nervous system. As MNs die, patients progressively lose their ability to control voluntary movements, become paralyzed and eventually die from respiratory/deglutition failure. Despite the selective MN death in ALS, there is growing evidence that malfunctional astrocytes play a crucial role in disease progression. Thus, transplantation of healthy astrocytes may compensate for the diseased astrocytes.

**Methods:**

We developed a good manufacturing practice-grade protocol for generation of astrocytes from human embryonic stem cells (hESCs). The first stage of our protocol is derivation of astrocyte progenitor cells (APCs) from hESCs. These APCs can be expanded in large quantities and stored frozen as cell banks. Further differentiation of the APCs yields an enriched population of astrocytes with more than 90% GFAP expression (hES-AS). hES-AS were injected intrathecally into hSOD1^G93A^ transgenic mice and rats to evaluate their therapeutic potential. The safety and biodistribution of hES-AS were evaluated in a 9-month study conducted in immunodeficient NSG mice under good laboratory practice conditions.

**Results:**

In vitro, hES-AS possess the activities of functional healthy astrocytes, including glutamate uptake, promotion of axon outgrowth and protection of MNs from oxidative stress. A secretome analysis shows that these hES-AS also secrete several inhibitors of metalloproteases as well as a variety of neuroprotective factors (e.g. TIMP-1, TIMP-2, OPN, MIF and Midkine). Intrathecal injections of the hES-AS into transgenic hSOD1^G93A^ mice and rats significantly delayed disease onset and improved motor performance compared to sham-injected animals. A safety study in immunodeficient mice showed that intrathecal transplantation of hES-AS is safe. Transplanted hES-AS attached to the meninges along the neuroaxis and survived for the entire duration of the study without formation of tumors or teratomas. Cell-injected mice gained similar body weight to the sham-injected group and did not exhibit clinical signs that could be related to the treatment. No differences from the vehicle control were observed in hematological parameters or blood chemistry.

**Conclusion:**

Our findings demonstrate the safety and potential therapeutic benefits of intrathecal injection of hES-AS for the treatment of ALS.

**Electronic supplementary material:**

The online version of this article (10.1186/s13287-018-0890-5) contains supplementary material, which is available to authorized users.

## Background

Amyotrophic lateral sclerosis (ALS) is an adult-onset disease characterized by the loss of both upper and lower motor neurons (MNs). Symptoms include progressive paralysis of MN target muscles. The disease is incurable, and fatal within 3–5 years of first symptoms, due to respiratory failure when the diaphragm is affected [1]. About 10–15% of cases of ALS are familial, and the other cases are sporadic. Familial ALS includes mutations in Cu^2+^/Zn^2+^ superoxide dismutase-1 (SOD1) [2] and in RNA/DNA binding proteins FUS and TAR DNA binding protein-43 [3]_._ However, the most frequent genetic cause of ALS (40% of familial ALS) is an amplification of a hexanucleotide in a noncoding region of the *C9orf72* gene [4].

The pathological mechanisms for ALS are still not well understood and the proposed mechanisms include inflammation, oxidative stress, glutamate cytotoxicity and protein aggregation. Although MNs are the main affected cells in the disease, a growing body of evidence suggests the involvement of astrocytes in the pathology of ALS in a non cell autonomous pathway. The contribution of astrocytes to the pathology of ALS is probably a combination of loss of homeostatic functions and/or gain of toxic functions. Several mechanisms by which ALS patients’ astrocytes affect ALS pathology include astrocyte toxicity; astrocytes that were isolated from sporadic and familial postmortem ALS patients and astrocytes derived from iPSCs of ALS patients have been shown to be toxic to healthy (WT) MNs [5, 6]. Similar results were obtained by primary astrocytes isolated from the hSOD1^G93A^ mouse model with both WT and MNs derived from ALS [7, 8]. The toxic effect of astrocytes on MNs was also demonstrated by addition of astrocyte condition medium [9, 10]. This lead to the notion that astrocytes of ALS patients secrete toxic/mutated proteins that cause specific death of MNs. This hypothesis is also supported by in-vivo studies in the hSOD1^G93A^ high copy number ALS models [11–14]. Another proposed mechanism is the reduction of functional astrocytic glutamate uptake suggested to contribute to glutamate excitotoxicity found in ALS patients [15]. GLT-1, a glutamate transporter (aka EAAT2), was found impaired in ALS patients [16, 17]. In-vivo studies have demonstrated that focal loss of GLT-1 in the ventral horn of the spinal cord precedes disease onset in a transgenic rat model for ALS overexpressing SOD1 [18]. Transplantation of SOD1(G93A) glial-restricted precursor cells–glial progenitors that are capable of differentiating into astrocytes in the cervical spinal cord of WT rats induced host MN ubiquitination and death, forelimb motor and respiratory dysfunction, and reactive astrocytosis and reduced GLT-1 transporter expression in WT animals [11].

Inflammation-mediated neuronal injury is also recognized as a major factor to promote ALS disease progression and amplifies MN death-inducing processes. The neuroimmune activation is not only a physiological reaction to cell-autonomous death, but also an active component of non-autonomous cell death. Astrocytes participate in the cellular response to damage and danger signals by releasing inflammation-related molecules like NO, IL-6, INF-γ, Prostaglandin D2, TGF-β and TNF-α that can induce the apoptosis of neurons observed in ALS disease [19–23]. In both physiological and pathological conditions, astrocytes secrete a wide range of factors with multiple influences on their cellular neighbors.

In addition, disruption of the astrocytic TNFR1–GDNF axis accelerates MN degeneration and disease progression, as the levels of the protective agents for MNs, glial-derived neurotrophic factor (GDNF), are reduced [24]. Astrocytes in the ALS rat model acquire an accelerated senescent phenotype that shows reduced support in MNs, that can be partially reversed by GDNF [25]. Another factor that plays a role in ALS pathology is vascular endothelial growth factor (VEGF), originally described as a factor with a regulatory role in vascular growth and development but it also directly affects neuronal cells [26, 27]. Transgenic mice expressing reduced levels of VEGF develop late-onset MN pathology, similar to that of ALS [28, 29]. VEGF is secreted by astrocytes and has been shown to protect MNs from excitotoxic death, as occurs in ALS [30]. In line with these results, low levels of VEGF and GDNF were reported in the cerebrospinal fluid (CSF) of ALS patients [31]. Other mechanisms include activation of necroptosis [32] and mitochondrial alterations [33–37].

These observations led to the rationale that ALS could be treated by implantation of normal wild-type healthy astrocytes from an external source, to support or replace dysfunctional ALS astrocytes [38]. In the present work, we have used human embryonic stem cells (hESCs) as a source for large-scale production of astrocyte progenitor cells (APCs), which can be stored as frozen banks. These APCs can be further expanded and differentiated into an enriched population of young committed astrocytes by removal of the growth factors for 7 days (hES-AS), which demonstrate functional properties of “healthy” astrocytes in vitro. These properties include: uptake of glutamate; production and secretion of a wide diversity of neuroprotective factors, as seen by secretome analysis; promotion of axonal outgrowth; and protection of MNs from oxidative stress. In animal ALS models (high-copy number hSOD1^G93A^ transgenic mice and rats), we show that intrathecal injection of hES-AS into the CSF of hSOD1^G93A^ mice and rats had significant effects on delaying disease onset, maintaining motor performances and delayed death. To obtain safety data that are relevant to both hES-AS and to their proposed clinical use, we conducted long-term safety and toxicology studies in NSG immune-deficient mice. These studies were designed to address key safety aspects associated with direct administration of hES-AS into the CSF by intrathecal injection, including toxicity, biodistribution, long-term engraftment and formation of tumors.

## Results

### Direct differentiation of hESCs into astrocyte progenitor cells and young astrocytes

**Fig. 1:**
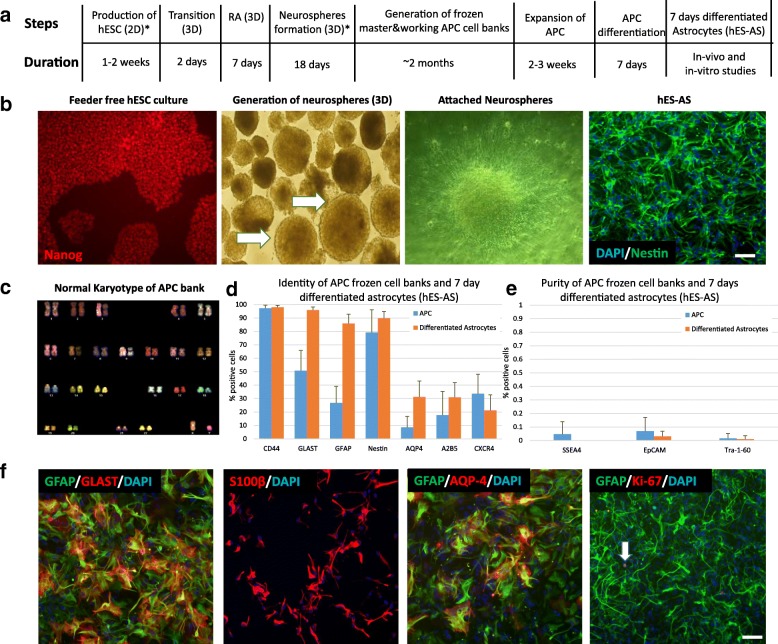
Differentiation of human embryonic stem cells into astrocyte progenitor cells and committed astrocytes. **a** Steps and timeline for differentiation of hESCs first into astrocyte progenitor cells (APCs) which can be stored frozen in APC banks. These APCs are further expanded with growth factors (bFGF, EGF and human serum), and then differentiated into astrocytes (hES-AS) by removal of growth factors for 7 days. **b** Representative images of different steps from hESCs to APCs (as in **a**, steps marked by asterisk). Arrows show selected neurospheres. **c** Representative spectral karyotyping analysis showing normal karyotype of APC cell bank at passage 12. **d** Flow cytometry analysis on nine batches of APC banks (grown with human serum, bFGF and EGF) versus 13 batches of astrocytes differentiated for 7 days showing expression of astrocytic markers (CD44, GLAST, GFAP, and Aquaporin-4) and neuroepithelial stem cell markers (Nestin, A2B5 and CXCR4). **e** Flow cytometry analysis of APCs and astrocytes differentiated for 7 days (same batches as in **d**) showing very low expression of pluripotent markers (below limit of detection, 0.1%). **f** Representative immunofluorescence images of astrocytes differentiated 7 days, showing expression of astrocyte markers (GFAP, GLAST, S100β and AQP-4) and very low proliferation marker (Ki-67, arrow). Scale bars = 100 μm. Error bars represent SD. hESC human embryonic stem cell, DAPI 4′,6-diamidino-2-phenylindole, GFAP Glial Fibrillary Acidic Protein, GLAST Glutamate Aspartate Transporter, RA Retinoic acid

Two hESC lines (HADC100 and NCL-14) were used to produce astrocytes for engraftment in hSOD1^G93A^ ALS animal models. Both hESC lines had a normal karyotype, expressed pluripotency markers and were capable of differentiating into all three embryonic germ layers [39, 40]. We modified our previously reported protocol [41] to generate an enriched population of APCs from hESCs, followed by further differentiation of the APCs into functional astrocytes (Fig. 1a). The protocol was optimized to include good medical practice (GMP)-grade materials and factors to be compatible for clinical use. In brief, hESC cultures having at least 70% of pluripotent stem cells expressing the SSEA4, TRA-1-60 and EPCAM markers were used as a starting material. The hESCs were detached and cultured in suspension with stepwise changes in media composition (Fig. 1a, b). First, all-*trans* retinoic acid and EGF were added for 7 days. This elicited increased production of bone morphogenetic factors (i.e. BMP4, BMP6, BMP2, BMP7 and BMP11), which were found to be essential for obtaining glial restricted cells, particularly astrocyte lineage cells [41, 42]. The suspension culture was continued with EGF resulting in the formation of neurospheres, which were seeded in 2D culture on laminin. The cells were expanded by successive passages in the presence of growth factors (bFGF and EGF) and human serum with the doubling time being 21 ± 2.6 h. This produced APCs that can be stored as frozen cell banks. The APC karyotype was tested at different passages (up to passage 12) and was found normal (Fig.1c). Flow cytometry analysis of APCs showed that the levels of pluripotent markers, SSEA-4, EPCAM and Tra-1-60, were < 0.2% (Fig. 1e). Above 90% of APCs were positive for the astrocytic marker CD44 [43] (Fig. 1d). The APCs had additional astrocytic markers such as the Glutamate Aspartate Transporter (GLAST, aka Excitatory Amino Acid Transporter 1 (EAAT1)) [44], glial fibrillary acidic protein *(*GFAP) [45] and Aquaporin-4 (AQP-4) [46], as well as neuroepithelial stem cell markers Nestin, A2B5 and CXCR-4 (Fig. 1d). The frozen/thawed APCs were further expanded for 2–3 weeks and then differentiated toward committed astrocytes, by removing growth factors EGF and bFGF as well as human serum from the media and adding vitamin C. After 7 days without growth factors (7-day astrocytes, hES-AS), flow cytometry showed that the percentages of GLAST, GFAP and AQP-4 astrocytic markers were increased compared to APCs (Fig. 1d). Upon differentiation of APCs toward committed young astrocytes there were no remaining undifferentiated cells, as shown by the levels of TRA-1-60, SSEA-4 and EPCAM, which remained < 0.1% (Fig. 1e), indicating high purity and low risk of teratoma formation [47]. It is important to note that only few Ki-67-positive cells were observed in hES-AS cultures (Fig. 1f), indicating that most hES-AS are post mitotic.

### Biological functionality of hES-AS

#### Glutamate uptake capacity

**Fig. 2:**
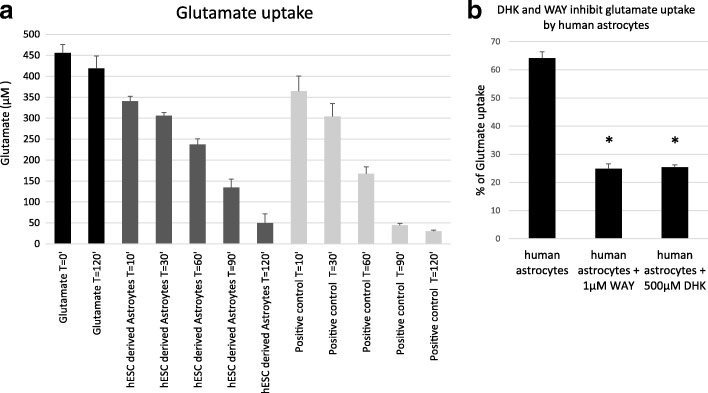
hES-AS take up glutamate from medium. **a** Glutamate concentration measured in solutions with 500 μM glutamate that were incubated for indicated times either alone (black bars 1–2) or with hES-AS differentiated for 28 days (black bars 3–7). Kinetics of glutamate removal by hES-AS similar to that by astrocytes extracted from human spinal cord (gray bars). **b** Percentage of glutamate uptake after 60 min by hES-AS alone or in presence of inhibitors of glutamate transporter GLT-1, WAY-213,613 (1 μM) and DHK (500 μM). Error bars are SD of triplicates. *p < 0.05. hESC human embryonic stem cell, DHK dihydrokainic acid

The glutamate uptake capacity of hES-AS was tested by incubating the cells in medium containing 0.5 mM glutamate and measuring the remaining concentration of the neurotransmitter at different times up to 120 min. Astrocytes from human spinal cord served as positive control and medium without cells as negative control. As shown in Fig. 2a, the hES-AS take up glutamate from the medium occurred in a time-dependent manner similar to the control human spinal cord astrocytes. After 2 h, more than 85% of the glutamate was removed from the culture media.

To investigate whether GLT-1 (EAAT2) participates in the glutamate uptake, the same experiment was done in the presence of either WAY-213,613 (1 μM) or dihydrokainic acid (DHK, 500 μM) [48]. With either of these GLT-1 inhibitors (Fig. 2b) the removal of glutamate in 60 min was inhibited by 60% (from 64.1% removal in the control to 25% with the inhibitors), demonstrating that a significant part of the glutamate uptake can be attributed to GLT-1 activity in the hES-AS.

#### Neuroprotective effect against oxidative stress

**Fig. 3:**
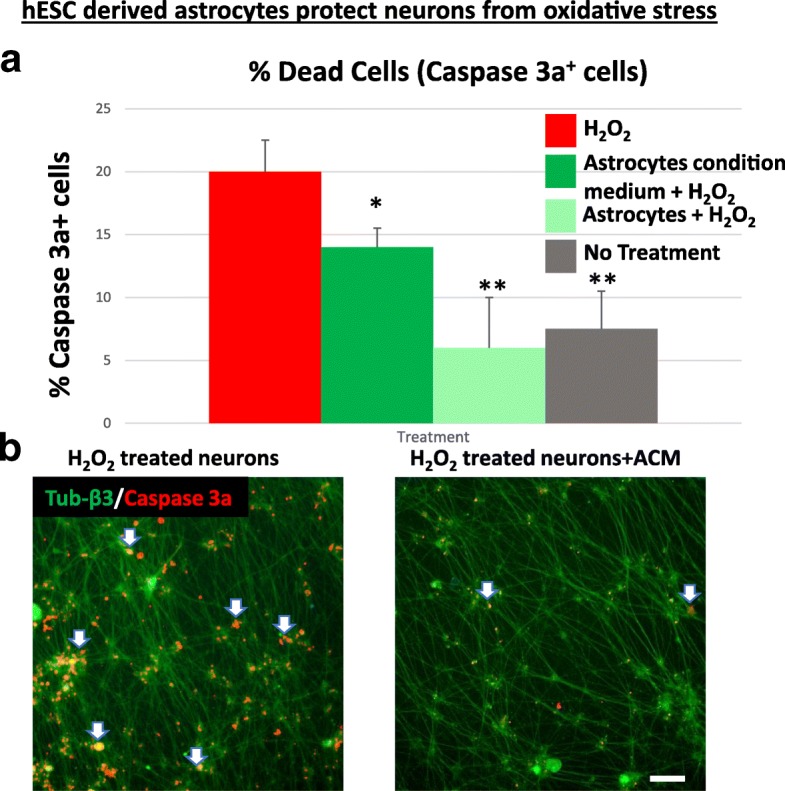
hES-AS protect MNs from oxidative stress. **A** Mouse motor neurons exposed in 96-well plates to 150 μM H_2_O_2_ for 6 h (bar 1) or left untreated (bar 4). During H_2_O_2_ treatment, neuron cultures supplemented with conditioned medium from hESC-derived astrocytes, differentiated for 28 days (ACM, bar 2), or with 20,000 of the same hES-AS (bar 3). After fixation, cells double-stained with anti-tubulin β3 antibody (neuron marker, green) and anti-Caspase-3a (apoptotic marker, red). Percentage of apoptotic neurons (Caspase3a over tubulin β3-positive cells) counted using high-content image screening system (Arrayscan; Cellomics). Results represent average ± SD for 10 wells of 96 well-plate per treatment (for each well, 49 fields were analyzed). *p < 0.05; **p < 0.01. **b** Left panel: representative image of neuron cultures with H_2_O_2_ treatment. Apoptotic neuronal cell bodies yellow (arrows, due to overlapping of red Caspase-3 staining with green tubulin β3). Right panel: with ACM, much less apoptotic yellow cells are seen. Scale bar: 100 μm. hESC human embryonic stem cell, H_2_O_2_ hydrogen peroxide

Cultures of mouse spinal cord MNs were challenged with 150 μM hydrogen peroxide (H_2_O_2_). The number of apoptotic MNs was measured after staining for activated caspase-3 and the total number of MNs being measured by staining for tubulin-β3. Using high-content image screening analysis, we calculated the percentage of apoptotic MNs (seen as yellow cells, Fig. 3b, left panel). The results (Fig. 3a) indicate a significant decrease (*p* < 0.05) in MN death by adding conditioned medium from the hES-AS, as seen by the decrease in caspase-3-positive cells (Fig. 3b, right panel). When the hES-AS were added in coculture with the MNs, there was a greater decrease in apoptosis resulting from oxidative stress (Fig. 3a, *p* < 0.01) to levels similar to spontaneous apoptosis. These results demonstrate the neuroprotective effects by hES-AS in vitro.

#### hES-AS stimulate axonal outgrowth in vitro

**Fig. 4:**
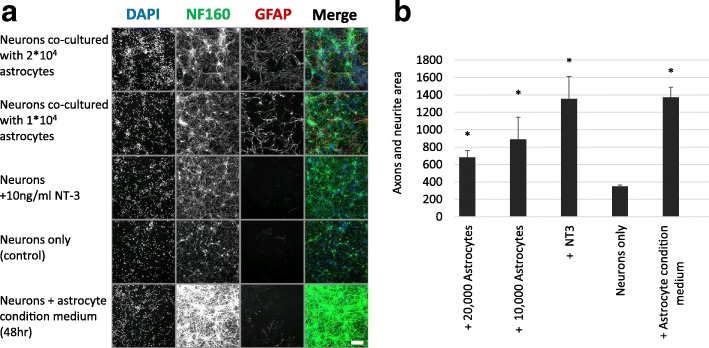
hES-AS and their conditioned medium stimulate axonal outgrowth in cortical neurons. **a** Mouse cortical neurons cocultured with hES-AS (7-day differentiated APC) (2 × 10^4^ and 4 × 10^4^ cells), or with neurotrophin 3 (NT3) as positive control, or left untreated (negative control). Last row shows neurons cultured with conditioned medium from same hES-AS (taken after 48 h of culture). Representative images of cells stained with DAPI and by immunofluorescence for neurofilament-160 (NF160) and GFAP shown for each condition. Stimulation of axon and neurite outgrowth seen from NF160 stain and merge of NF160 (green) and GFAP (red). Scale bar = 100 μm **b** By high-content image screening analysis (Arrayscan; Cellomics), area covered by axon and neurite outgrowth quantified, using 49 fields for each of six replica wells from each experimental conditions. Error bars represent SD. *Student’s *t* test, *p* < 0.05). DAPI 4′,6-diamidino-2-phenylindole, GFAP Glial Fibrillary Acidic Protein

We next assessed the ability of hES-AS to induce axonal outgrowth in vitro. Rat primary cortical neurons derived from day 18 embryos were precultured for 2 days in Neurobasal medium (with B27) and then further cultured for 4 more days in either medium alone or supplemented with 10 ng/ml Neurotrophin-3 (NT-3, as positive control), or cocultured with hES-AS (1–2 × 10^4^ cells), or cocultured with hES-AS conditioned medium (collected from days 5 to 7 of astrocyte differentiation). The cultures were labeled by ICF with antibodies against axonal neurofilament-160 and GFAP markers. Representative images of the five conditions are shown in Fig. 4a. By high-content image screening analysis, the total area of axons and neurites in the NF160-stained images was determined. A significant increase in axonal outgrowth was seen in the neurons cocultured with hES-AS (Fig. 4b, *p* < 0.01). Moreover, addition of the hES-AS conditioned medium was found to stimulate axonal outgrowth to a similar extent as compared to the cocultures, indicating that this neurogenic activity can be attributed to factors secreted by these astrocytes. As expected, GFAP-positive cells were observed only in the cocultures, indicating that the rat cortical neurons were not contaminated by rat astrocytes.

#### Neurotrophic factor synthesis and secretion

We first measured the levels of known neurotrophic factors GDNF, BDNF, VEGF and IGF-I both in hES-AS culture supernatant media and in cell extracts (cell content). VEGF was found to be secreted from hES-AS that were differentiated without growth factors for 28 days (Additional file 1: Figure S1). IGF-1 was also secreted, whereas GDNF and BDNF were found inside the cells but less was secreted (Additional file 1: Figure S1). The levels of these classical neurotrophic factors were in the range found in human CSF [49, 50].

To have a more comprehensive view of the factors secreted by 7-day and 28-day differentiated hES-AS, we carried out secretome analysis. The 48-h conditioned medium of replica cultures of hES-AS were analyzed using the human Quantibody Kiloplex Array (RayBiotech), capable of detecting 1000 proteins. A total of 220 protein factors were found to be secreted at levels over the threshold in 7-day hES-AS, about 25% of which being more abundant at 28 days (see Additional file 2: Table S1). Among the highest 120, there were 25 proteins with activities in neurogenesis, axon or neurite outgrowth or axon guidance. Interestingly, there were 13 proteins with antiprotease activity. In addition, there were extracellular matrix (ECM) components, cell adhesion membrane proteins and a few peptidases. This indicates that there is a complex set of factors secreted by the hES-AS, beyond the classical neurotrophic factors. Many of these factors may be responsible for the neurogenic and neuroprotective activities observed earlier. Examples of the secreted factors with effects on neurons or with antiprotease activity are presented in Table 1. Several of these factors may be relevant for potential therapeutic mechanism of action in ALS (e.g. Osteopontin, tissue inhibitor of metalloproteinase (TIMP)-1 and TIMP-2, Midkine, MIF; see Discussion).

**Table 1:** hES-AS secrete a variety of factors with effects on neurons or with antiprotease activity

	7-day astrocytes (ng/ml/10^**6**^ cells)	28-day astrocytes (ng/ml/10^**6**^ cells)
Secreted factors with effects on neurons		
Osteopontin (OPN)	53.1 ± 29	56.8 ± 5.5
Dickkopf-3 (DKK-3)	43.1 ± 14.2	33.8 ± 1.6
Thrombospondin (TSP-1)	22.7 ± 11.5	118.9 ± 36.8
Secreted Frizzled Protein (sFRP3)	20.8 ± 10.9	41.2 ± 23.0
Brevican proteoglycan	15.6 ± 4.9	12.6 ± 3.3
Tripeptidyl peptidase (CLN2)	11.5 ± 4.2	20.1 ± 11.7
Clusterin	9.5 ± 3.2	6.5 ± 0.5
Midkine	8.4 ± 3.0	6.1 ± 3.5
NSE	3.5 ± 1.8	0.9 ± 0.2
MIF chemokine	1.8 ± 0.6	0.4 ± 0.1
CXCL16	1.5 ± 0.8	2.1 ± 0.2
Thrombospondin-2	0.85 ± 0.4	2.3 ± 0.4
GRFα-1	0.45 ± 0.2	1.0 ± 0.6
VEGF	0.05 ± 0.02	0.23 ± 0.09
Antiprotease activity		
Fetuin A	1816.0 ± 677	1404.7 ±+ 129.4
Tissue inhibitor of metalloprotease TIMP-2	16.6 ± 6.8	14.5 ± 0.8
PAI-1 Serpine 1 protease inhibitor	7.2 ± 6.2	54.9 ± 5.9
Tissue inhibitor of metalloprotease TIMP-1	7.0 ± 3.8	6.5 ± 0.8
Serpin A4	4.3 ± 2.5	4.0 ± 0.4

Results shown as mean ± standard deviation for triplicates of hES-AS differentiated for 7 days and duplicates of hES-AS differentiated for 28 days

Secretome analysis performed on 48-h conditioned media of hES-AS. Listed are factors with activities in neuroprotection, neurogenesis, axon growth or guidance, as well as antiproteases. For relevance to amyotrophic lateral sclerosis, see Discussion. Complete secretome list presented in Additional file 2: Table S1

*GRF* GDNF family receptor, *hES-AS* human embryonic stem cell-derived astrocytes (differentiated from APCs for 7 days), *MIF* macrophage migration inhibitory factor, *NSE* neuron specific enolase, *PAI* plasminogen activator inhibitor, *VEGF* vascular endothelial growth factor

### Transplantation of hES-AS in SOD1^G93A^ mouse and rat ALS models

Both SOD1^G93A^ mouse and rat models present a typical pattern of ALS disease progression, in which onset of the disease in hindlimbs precedes that in forelimbs, and in which the end stage results from compromised respiratory function [18, 51]. A dose of 2 × 10^6^ hES-AS (differentiated for 7 days) were injected into the CSF of hSOD1^G93A^ mice through the cisterna magna (CM), either once on day 67 ± 2 after birth or twice on days 67 ± 2 and 97 ± 2 (Additional file 3: Figure S2). Disease onset was determined by the loss of 3% of maximal body weight. Results demonstrate that double transplantation of the hES-AS significantly delayed disease onset compared to sham-injected controls (Additional file 3: Figure S2A; median 119 days vs 112 days; *p* = 0.0012, log-rank), and was better than with a single injection. Motor performance, as measured by Rotarod test as well as by neurological scoring, was significantly improved in mice that were injected twice with hES-AS, compared to sham-injected mice (Additional file 3: Figure S2D, E; *p* < 0.05). Two injections were better than a single dose. The survival of mice injected twice with hES-AS was somewhat prolonged compared to sham-injected mice (Additional file 3: Figure S2B; median survival 130 days vs 126.5 days; but *p* = 0.1, log-rank). With the double injection there was also a trend for longer survival at late times, compared to one injection.

**Fig. 5:**
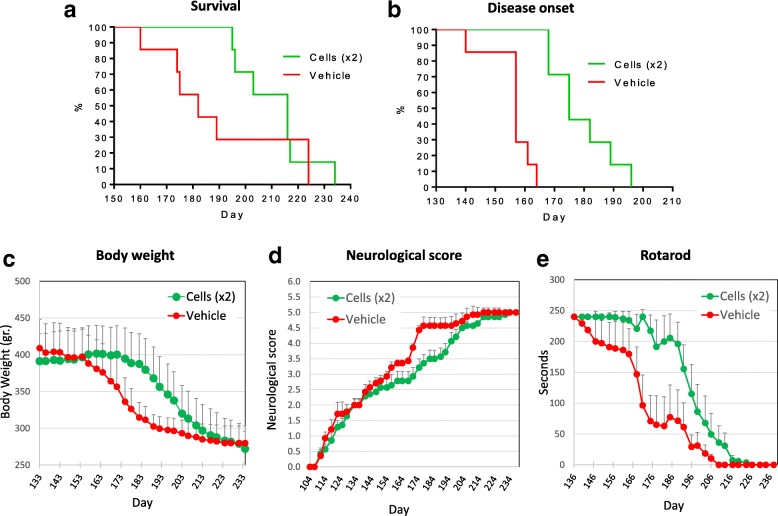
Effect of hES-AS transplantation on disease onset, motor activity and survival in hSOD1^G93A^ rat ALS model. hES-AS (APCs differentiated for 7 days) injected intrathecally through lumbar puncture (L5–L6), in two doses of 3 × 10^6^ cells each on days 50 and 70 after birth in hSOD1G93A rats. **a** Kaplan–Meir survival curves of rats treated with hES-AS (green) show prolongation of median survival compared to sham-injected group (vehicle, red). **b** Kaplan–Meir plot of disease onset (defined by 3% body weight loss) shows significant delay in hES-AS-treated ALS rats. **c** Body weight maintained significantly longer in hES-AS-treated ALS rats. **d** Neurological score. **e** Significant prolongation of motor performance on Rotarod in hES-AS-treated ALS rats. Same seen by grip strength measurement. **c, d** Values represent mean ± SEM

We then shifted to the rat hSOD1^G93A^ ALS model, which allows use of intrathecal injection by lumbar puncture (LP), a route of administration similar to what could be applied in human patients. The rat model also allowed administration of more cells. A total of 6 × 10^6^ hES-AS (differentiated for 7 days) was administered divided into two injections, the first on day 50 ± 2 after birth and the second on day 70 ± 2. A control group was sham-injected with the vehicle solution. The LP injections were in the subarachnoid space between L5 and L6 vertebra. The median survival of the hES-AS-treated rats was 216 days compared to 182 days in the sham-injected rats (Fig. 5a); Kaplan–Meier analysis for the entire experiment showed an increased survival trend (*p* = 0.077 by area under the curve (AUC) analysis). The disease onset was delayed very significantly by hES-AS treatment (Fig. 5b, *p* = 0.0001); Kaplan–Meier analysis showed that 50% of treated rats lost 3% of their body weight by day 175 compared to day 157 in the sham-injected group. The hES-AS-treated rats maintained their body weight significantly longer (by about 30 days) than sham-injected rats (Fig. 5c, *p* = 0.007). A set of motor tests demonstrated the therapeutic effect of the hES-AS treatment. First the overall development of clinical symptoms, as evaluated by open field neurological scoring, was significantly delayed (Fig. 5d, *p* < 0.001). The decline of motor functionality, as measured by “time to fall” from a Rotarod, was markedly slowed down by hES-AS treatment, the animals maintaining normal motor activity for more than 1 month longer than the controls (Fig. 5e, *p* < 0.001). Likewise, the loss of forelimb muscle strength, as measured by the grip strength test, was significantly slowed down, just as the Rotarod performance (*p* < 0.001; data not shown). Other observations were that no tumors were observed in the animals post mortem.

### Assessment of safety, tumorigenicity and biodistribution of hES-AS following a single injection to the cisterna magna of NSG mice

**Fig. 6:**
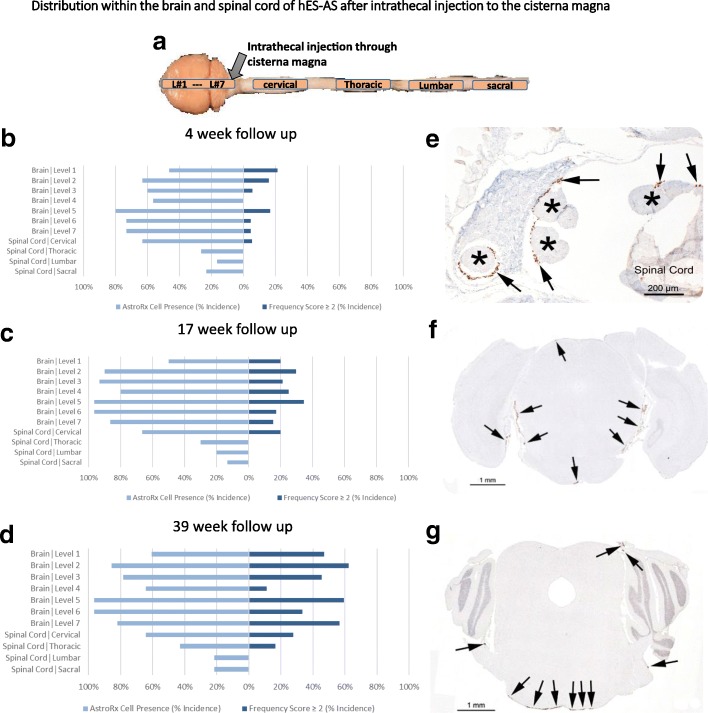
hES-AS distribute throughout CNS after intrathecal injection. hES-AS (400,000 cells) differentiated for 7 days transplanted intrathecally into NSG mice (into CSF through CM). **a** Illustration of brain and spinal cord sections performed: seven brain sections (L#1–L#7 as in [64]) and four of representative regions of spinal cord. **b–d** Graphical representation of AstroRx cell presence (as determined by Alu^+^ cell staining) and percent incidence of frequency scores ≥ 2 (one to three foci of 10–20 cells per foci) after 4-week (**b**), 17-week (**c**) and 39-week (**d**) follow up. AstroRx Cell presence calculated as incidence (%) from all samples (*n*) within each group. Frequency of score ≥ 2 calculated as incidence (%) of frequency scores ≥ 2 from only those sections in which AstroRx cells present. **e–g** Representative images of different sections demonstrating distribution of hES-AS throughout CNS using ISH with and *Alu Y* probe (human specific) of 17-week cohort. **e** Sacral region of spinal cord with numerous Alu^+^ cells (arrows) along surface of the spinal nerves (asterisks). **f** Brain, level 5. Arrows indicate cells along meningeal surface at many locations. **g** Brain, level 6. Arrows indicate Alu^+^ cells along meningeal surface along base of medulla at brain level 6. Cells attached to the pia mater (arrows). hES-AS human embryonic stem cell-derived astrocytes (differentiated from APCs for 7 days)

The safety, tumorigenicity and biodistribution phases were conducted in compliance with principles of good laboratory practice (GLP) over a period of up to 9 months. hES-AS, differentiated for 7 days, were injected intrathecally into the CSF of NSG mice through the CM with 0.4 × 10^6^ cells/ mouse, or with a vehicle. Mice were sacrificed 4, 17 and 39 weeks post transplantation. No clinical signs were attributable to treatment during the monitoring periods. Cell-injected mice made similar body weight gain by 4, 17 and 39 weeks post dose to the vehicle control groups. In addition, there were no differences from the vehicle control at the hematological and blood chemistry investigations at 4, 17 and 39 weeks after dose administration (data not shown). Histopathological evaluation of the brain and spinal cord was performed to assess tumorigenicity. No teratoma or other tumors that could be related to the treatment were seen in the transplanted animals in any of tested time points. In order to evaluate the hES-AS distribution in the CNS, the sections were stained using an in-situ hybridization (ISH) technique with a human-specific *Alu Y* sequence. Cells positive for *Alu Y* sequences were present at all levels of the CNS in similar incidences between the three study time points. The incidence for the various levels range between 17% (distal areas from injection site) and 80% (at vicinity of the injection site) after 4 weeks, between 13% and 97% after 17 weeks and between 21% and 96% after 39 weeks (Fig. 6 and Additional file 4: Table S2). The cells were almost uniformly seen along the meninges, attached to the pia mater. To assess the biodistribution of hES-AS outside the CNS, the detection of human cells in mouse tissues was performed by quantitative real-time PCR (qPCR), targeting the specific sequence of the human *Alu* sequence. The detection was performed in nine organs including the spleen, kidney, testis/ovary, liver, heart, bone marrow of the femur, lungs, and cervical lymph nodes. The qPCR method was validated prior to the study and both the limit of detection (LOD) and the limit of quantification (LOQ) were set at one human cell (DNA equivalent) per 1 μg of mouse DNA. The PCR results showed no detection of human DNA above the LOD in any of the tested organs 4 and 17 weeks after transplantation.

**Fig. 7:**
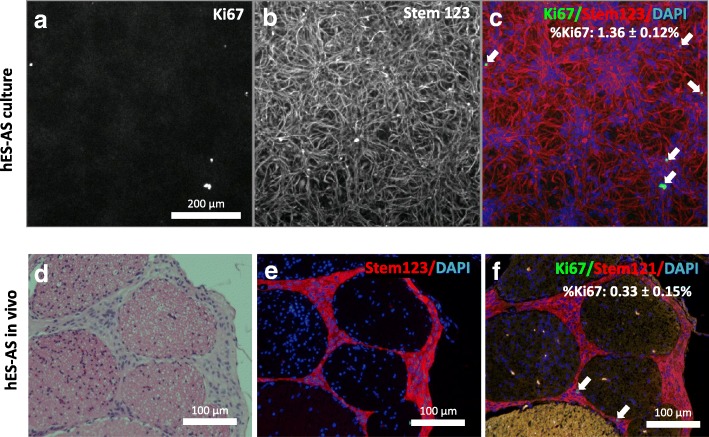
hES-AS are post mitotic and maintain their astrocytic identity in vivo. **a**–**c** High-content analysis of hES-AS cells in vitro displayed homogeneous expression of human GFAP (Stem123). %Ki67^+^ cells calculated as % Ki67^+^ nuclei / total number of nuclei. Ki67^+^ cells rarely found within hES-AS cell population (arrows). **d**–**f** Two million hES-AS injected intrathecally into the lumbar region twice, with interval of 21 days. Analysis of graft, 8 weeks post first cell injection, showed transplanted cells were located in subarachnoid space, attached to pia mater of lumbar spinal cord and nerve bundles. Cells maintained their astrocytic characters and homogeneously expressed human-origin GFAP. %Ki67^+^ hES-AS cells calculated as % Ki67^+^ nuclei / total number of nuclei of Stem123^+^ cells. Ki67^+^ staining very rare among hES-AS cells (arrows), indicating that cells are non-proliferative in vivo. hES-AS human embryonic stem cell-derived astrocytes (differentiated from APCs for 7 days), DAPI 4′,6-diamidino-2-phenylindole

We also examined the astrocytic identity of hES-AS in vivo 2 months after their transplantation in the CSF of immunodeficient mice. Histological sections were stained for the general human cytoplasmic specific marker Stem121 and for Stem123 (human-specific GFAP antibody) in order to ascertain the presence of human cells. All Stem121-positive cells were positive for human GFAP, demonstrating that the transplanted hES-AS maintained their astrocytic identity in the CSF (Fig. 7). Further staining for the cell cycle marker Ki67 showed that 0.33 ± 0.15% of Stem121-positive cells in the CSF were also positive for Ki67, indicating for the very low proliferative capacity of hES-AS in vivo (Fig. 7g).

## Discussion

This work describes the derivation of young astrocytes from human embryonic stem cells (hES-AS), which have therapeutic activity in vivo following intrathecal injection into the CSF of transgenic SOD^G93A^ rats and mice. In addition, we describe the results of a preclinical safety study in immunodeficient mice to assess the tumorigenicity potential and biodistribution of hES-AS in target and distal organs.

To date, two FDA-approved drugs, riluzole and Radicava, were shown to modestly attenuate motor deterioration in ALS patients [52–55]. Still, many late-phase clinical trials failed to demonstrate a significant improvement in slowing down disease progression when using single-target drugs [56]. ALS is a multifactorial disease and therapeutic approaches should take into account the multiplicity of mechanisms that underlie MN degeneration in this disease. Thus, a potential therapy that acts through multiple mechanisms of action to treat the broad pathological aspects of the disease is more likely to be effective. An example for the complexity of the disease is the involvement of astrocytes in the degeneration of MNs [5, 7, 8, 57]. Such noncell autonomous death of MNs caused by ALS-type astrocytes supports the rationale that transplantation of healthy human astrocytes into the CNS of ALS patients may compensate for the malfunctional astrocytes and rescue dying MNs (review in [38]).

hES-AS exhibit multiple activities that were shown to be impaired in ALS-type astrocytes. Astrocytes from ALS transgenic mice express more iNOS/NOS2, leading to increased release of NO, which exacerbates oxidative stress leading to MN death [58]. We show in our study that hES-AS protect in-vitro spinal cord MNs from oxidative stress produced by H_2_O_2._ In ALS patients, a decrease of the astroglial GLT-1 glutamate transporter is observed [16], leading to decreased glutamate uptake in the synaptic clefts of the spinal cord. Accumulation of excitatory glutamate makes MNs in ALS more susceptible to excitotoxicity [59]. hES-AS express both glutamate transporters GLAST and GLT-1 and efficiently uptake glutamate, which is in part due to their GLT-1 expression, as shown by GLT-1 inhibitors. Another mechanism by which the diseased astrocytes lead to MN death is by a decrease in the secretion of neurotrophic factors. hES-AS produce and secrete the neurotrophic factors GDNF, BDNF, IGF-1 and VEGF in a comparable amount to that of endogenous astrocytes. The neurotropic property of hES-AS was demonstrated by cocultures of hES-AS with neurons and by hES-AS conditioned medium alone, indicating activity of soluble secreted factors. Secreted VEGF is likely to play an important role by protecting neurons in ALS, reducing excitotoxicity [28, 60], and its concentration is lower in the CSF of ALS patients [31]. In addition, GDNF synergizes with VEGF to prolong survival in a murine ALS model [61]. Intrathecal injection of CSF from sporadic ALS patients to neonatal rats induces selective degeneration of MNs [62] and downregulates the levels of both BDNF and IGF-1 in the spinal cord [63]. Supplementation of BDNF reverses the neurodegenerative changes induced by ALS-CSF in MN cultures [64].

The nature of the secreted factors was further investigated by a secretome analysis, clearly illustrating the pleiotropic activity of the cells. hES-AS secrete many factors having activities on neurons [65, 66–68] as well as several antiproteases and factors which could remodel the ECM (see Table 1). Among the more abundant factors found in the secretome analysis, several have been linked to ALS, thereby shedding new light on the possible mechanisms of action underlying the observed therapeutic effect in ALS models. One of the most abundant factors in the secretome is Osteopontin (OPN/SSP1), which in the mutant SOD1 model of ALS is found to be associated with MNs that are more resistant to degeneration early in the disease, but low in the MNs more vulnerable to degeneration in ALS [69]. Conversely, the vulnerable MNs are high in matrix metalloproteinase MMP-9 (MMP9^high^ /OPN^low^), whereas MMP-9 is low and OPN is high in the ALS-resistant MNs [69, 70]. Exogenous addition of OPN has neurogenic effects, stimulating regeneration of motor axons [71] and protecting neurons after ischemia in vitro and in vivo [72]. Although MMP9 was not detected in the secretome of our astrocyte cultures, inhibitors of MMP9 and other matrix metalloproteases were abundantly secreted, particularly the tissue inhibitors of metalloproteases TIMP-1 and TIMP-2, which play a major role in preventing degradation of ECM components by MMPs or regulating ECM remodeling (review in [73]). Another chemokine found in the secretome is MIF, which has the capacity to save primary MNs from the degeneration caused by the ALS mutant SOD1 form, probably by acting as a chaperone [74]. Also secreted is Clusterin, another chaperone, promoting axon regeneration, as observed on peripheral sensory neurons [71], and increasing neuron survival [75]. Midkine secreted by astrocytes is a known neurotrophic factor promoting neurite outgrowth and neuron survival (review in [76]). The multiple nature of the factors secreted by the hES-AS supports a mode of action much more diversified than merely through the classical neurotrophic factors.

The efficacy of hES-AS to delay disease onset and to ameliorate disease progression was evaluated in transgenic high copy number SOD1^G93A^ mouse and rat models, which recapitulate many of the clinical symptoms of the ALS disease in humans [18, 51, 77]. Intrathecal injection of hES-AS significantly delayed the onset of the disease and slowed down the deterioration of motor function. These effects were more pronounced when the cells were administered twice (3–4 weeks apart) than with a single injection. Intrathecal injection into the CSF is in line with the proposed mode of action, in which the healthy astrocytes would work at a distance to modify the environment of brain and spinal cord MNs. Indeed, the CSF composition shows several changes in the course of ALS [78, 79], including an increase in oxidative stress markers, an increase in glutamate in at least 40% of patients and variations of VEGF concentration correlating with the length of survival [80], and other changes including OPN increase [81]. Moreover, the fact that inoculation of CSF from ALS patients to animals is neurotoxic [63] demonstrates that materials injected into the CSF can affect the parenchyma.

A major safety concern associated with pluripotent stem cell-based therapies is the presence of residual undifferentiated stem cells that might continue to divide without control or develop teratoma after their transplantation in the body [82, 83]. We minimize the possibility of teratoma formation by assuring a complete differentiation of hESCs into committed astrocytes with a normal diploid karyotype and minimal proliferation capacity. Teratoma formation from undifferentiated hESCs depends on several factors, among them the site of implantation and number of transplanted cells. Several studies reported that undifferentiated hESCs develop teratomas within 6 weeks after transplantation in immunodeficient mice [47, 82, 84, 85]. We previously reported that injection of undifferentiated hESCs intrathecally into immunodeficient mice results in teratoma formation within 5–7 weeks after injection [86]. In our current study, we evaluated the formation of teratomas, or any other tumor, by hES-AS up to 39 weeks after their intrathecal injection, long enough to allow development of teratomas. Histology evaluation showed the cells survived in the CSF for the entire duration of the study, attached to the pia mater along the neuroaxis, The cells uniformly expressed astrocytic markers with very rare coexpression of the cell cycle marker Ki67. Importantly, hES-AS did not develop teratoma or any other tumors in any of the treated mice. In line with these results, Priest et al. [87] also reported the absence of teratomas in the CNS following intraspinal injection of oligodendrocyte progenitors derived from hESCs into the spinal cord of immunodeficient rats.

To access the CNS, we chose the CSF as the injection site for hES-AS. The circulating CSF helps to distribute the injected cells throughout the subarachnoid space. In addition, injection into the CSF by LP is a common low-risk medical practice already demonstrated in several clinical trials with cell-based therapies [88–91]. A biodistribution evaluation of hES-AS in the CNS was performed by in-situ hybridization of the *Alu Y* gene at 4, 17 or 39 weeks following a single intrathecal injection of cells into immunodeficient mice. The analysis revealed the presence of hES-AS cells in the subarachnoid space throughout the entire CNS. Cell numbers were maintained stable over time, supporting that the cells remain quiescent in the CSF. The effective biodistribution of hES-AS along the entire CSF supports the clinical benefits we observed in SOD1^G93A^ models. We found an attenuation in motor activity loss in both lower and upper limbs and the tail, indicating that the cells exert their activity on multiple regions of the CNS. The possible migration of cells to distant organs was evaluated by qPCR for amplification of the *Alu Y* genomic sequence in nine organs. hES-AS were not found in any distant organ above the detection limit of the method (1 cell) at 4 and 17 weeks after their intrathecal injection. This confined distribution of the cells to the CNS minimizes any possible risk of presence of ectopic glial tissue in nontarget organs outside the CNS.

Large quantities of human astrocytes would be needed for the treatment of ALS patients worldwide. As shown here, clinical-grade human ESCs provide a robust and controlled source of cells for mass production of glial progenitors that can give rise to functional astrocytes. To comply with GMP standards, we adjusted our previous protocol, originally aimed to produce both astrocytes and oligodendrocytes [41], to include only GMP-grade materials. Under this protocol, large amounts of astrocyte progenitor cells (APCs) are obtained, which can be frozen in liquid nitrogen for long-term storage [41] as master and working cell banks for future expansion. Upon thawing of the APC vial, the differentiation into hES-AS is completed within 7 days of culturing. In terms of yield, using our protocol we can produce a total of 2 × 10^13^ hES-AS from a single batch of hESCs. Hence, the process described here is suitable for mass production of clinical-grade hES-AS per batch, which can potentially treat thousands of patients [92, 93].

In recent years, clinical trials of cell therapy in ALS have mainly used autologous transplantation of mesenchymal stem or stromal cells (MSCs) [89, 94], in which cells are taken from the patients and after in-vitro culture are returned to the same patient. While giving promising clinical efficacy, these autologous transplantations have limitations and it would be advantageous to develop allogeneic cells as a shelf-product that would provide a treatment for all ALS patients. Given that intrathecal administration is effective (as seen with the MSCs), it would be easier than injections in the spinal cord anterior horn, which requires major surgery as done in recent ALS clinical trials with neural stem cells taken from human organ donors [95, 96]. Future clinical trials could use human pluripotent stem cell cultures for mass production of neural cells, either from human iPSCs [97, 98] or from human ES cell lines as described here.

## Conclusions

Here we describe the derivation of a highly enriched population of functional, clinical-grade, human astrocytes (hES-AS) from embryonic stem cells. The hES-AS were shown to protect MNs by multiple mechanisms, similarly to normal astrocytes, including clearance of glutamate, secretion of multiple NTFs, neutralization of ROS and promotion of neural outgrowth. Intrathecal injection of hES-AS to rodent models of ALS delays disease onset, slows down disease progression and extends life expectancy. A 9-month safety study conducted in an immunodeficient NSG animal model, under GLP conditions, showed that intrathecal transplantation of hES-AS cells to the cerebrospinal fluid (CSF) is safe. Thus, these findings demonstrate the feasibility, safety and potential efficacy of intrathecal injections of hES-AS for the treatment of ALS. The safety and efficacy of hES-AS treatment in ALS patients will be tested in a phase I/IIa clinical trial (ClinicalTrials.gov identifier: NCT03482050).

## Methods

### Derivation of astrocyte progenitor cells and committed astrocytes from hESCs

Two clinical-grade hESC lines, were used: NCL14, licensed from the University of Newcastle; and HADC100, obtained from the Hadassah Medical Organization (HMO), Jerusalem (Prof. Benjamin Reubinoff). Master cell banks (MCB) and working cell banks (WCB) of these hESCs were created at Kadimastem Ltd. The undifferentiated state of the hESCs was routinely assessed by flow cytometry analysis of the surface markers SSEA-4, EpCAM and TRA-1-60, and by immunofluorescence staining for the transcription factors NANOG and OCT4. Both lines were propagated in undifferentiated state on a HFF feeder layer (25,000 cells/cm^2^) by passaging every 6–7 days using collagenase in order to detach the whole hESC colonies from the feeder cell layers. The colonies were mechanically broken and seeded in a ratio of 1:3–6. The hESCs were grown in ES1 media composed of KO-DMEM, 14% (v/v) KO serum replacement, 2 mM glutamine, 1× MEM nonessential amino acids, 0.1 mM β-mercaptoethanol and 25 U/ml penicillin, 25 μg/ml streptomycin (all from Life Technologies) and 8 ng/ml bFGF (R&D). Important to note is that for generation of clinical-grade hESCs, the cells were adapted to feeder free conditions and the media composition was changed to Essential 8™ (E8) medium (Thermo Fischer Scientific).

Formation of neurospheres (NS) was done in suspension (3D) cultures. In brief, the harvested hESC colonies were transferred into 100-mm ultralow attachment culture plates (Corning) containing ITTSPP/B27 medium. ITTSPP/B27 is a mixture of DMEM/F12 containing 1% B27 supplement, 1% Glutamax, 1.5% Hepes at pH 7.4 (all from Thermo Scientific), 1% penicillin/streptomycin/amphotericin solution (Biological Industries), 25 μg/ml human insulin (ActRapid; Novo Nordisk), 50 μg/ml human Apo-transferrin (Athens), 6.3 ng/ml progesterone, 10 μg/ml putrescine, 50 ng/ml sodium selenite and 40 ng/ml triiodothyronine (T3) (all from Sigma). ITTSPP/B27 was supplemented with 20 ng/ml r-human EGF (R&D Systems). After 2 days, the medium was switched to ITTSPP/B27 supplemented with 20 ng/ml EGF and 10 μM ATRA (Sigma). The culture was continued in suspension in the nonadherent plates for 7 days with daily replacement of the medium (stage 2; Fig. 1). During the last step, which allows for NS ripening, the culture was continued in ITTSPP/B27 medium supplemented with 20 ng/ml EGF for 18 days. Medium was replaced every other day (stage 3; Fig. 1). For APC expansion, round yellow NS were manually selected using a stereoscopic microscope and transferred into six-well plates coated with Matrigel or GMP-compliant laminin 521 (from Biolamina) in ITTSPP/B27 supplemented with 20 ng/ml EGF. Medium was replaced every other day for 7–10 days (passage 0). In order to produce a monolayer, the spheres were dissociated with TryplE (Thermo Scientific) and reseeded on ECM (passage 1) in N2/B27 medium consisting of DMEM/F12 with 0.5% (v/v) N_2_ supplement, 1% (v/v) B27 supplement, 1% Glutamax and 1.5% Hepes at pH 7.4 (all from Thermo Scientific). The growth factors EGF and bFGF (R&D Systems) were added at 10 ng/ml each. The monolayer cells were further passaged weekly until a sufficient number of cells was generated. Cells were then frozen in liquid nitrogen and stored as banks of APCs. Thawed APCs were further expanded as described earlier for 2–3 weeks. In order to differentiate the APCs toward astrocytes, EGF and bFGF were removed from the media, 50 μg/ml ascorbic acid (Sigma) was added and the culture was continued for 7 or 28 days.

### Immunocytofluorescence assays

Cells were fixed with 4% paraformaldehyde (PFA), washed with PBS and kept at 4 °C before staining. Permeabilization was done by 0.5% Triton X-100 in Blocking solution (5% BSA; Sigma) and 3% horse serum (w/v in PBS; Biological Industries). Incubation in the same blocking solution was done for 1 h at RT. Primary antibodies, diluted in blocking solution, were as follows: anti-Nanog, anti-Nestin (1:500; BD Pharmingen), anti-GFAP-cy3 (mouse monoclonal antibody (Mc), 1:500; Sigma), anti-GLAST (rabbit Mc, 1:100; Miltenibiotec), anti-S100 (rabbit polyclonal antibody, 1:100; DAKO), anti-AQP-4 (rabbit, 1:2000; Mc Abcam) and anti-Ki67 (rabbit, 1:50; Mc Cell Marque). After overnight incubation at 4 °C, secondary antibody (1:200; Jackson Immuno Research) was added for 1 h at RT, followed by the nuclear fluorescent dye DAPI (0.05 μg/ml; Sigma). Pictures were taken using Arrayscan VTI (Thermo Scientific, Cellomics).

### Immunohistochemical staining

Brain and spinal cord tissues were trimmed, decalcified and embedded in paraffin, sectioned at approximately 5 μm thickness and stained with hematoxylin and eosin (H&E). For immune-cytofluorescence assays, tissues were deparaffinized using the following washes: xylene (Sigma), two washes × 5 min; 100% ethanol, two washes × 5 min; 95% ethanol, one wash × 5 min; 70% ethanol, one wash × 5 min; and cold tap water, two washes × 5 min. Heat-induced epitope retrieval was performed by boiling the sections in a domestic microwave, twice for 10 min, using 100× H-3300 citrate-based solution (Vector Laboratories). Permeabilization was done by 0.5% Triton X-100 in blocking solution as described earlier, and incubation continued in the same blocking solution for 1 h at RT. Primary mouse Mc antibody Stem123 or Stem121 (1:500; Stem Cells) were added overnight and kept at 4 °C. Secondary antibody goat anti mouse Cy2 or Cy3 (1:200; Jackson Immuno Research) were added for 1 h at RT, followed by the nuclear fluorescent dye DAPI (0.05 μg/ml; Sigma).

### Karyotype

The test was performed using spectral karyotyping analysis (SKY) on cells from two APC banks (passages 11 and 12). The analysis was performed by the Stem Cell Core and Advanced Cell Technologies Unit, Department of Life Sciences Core Facilities, Weizmann Institute of Science.

### Flow cytometry

Cells were analyzed by flow cytometry for identity and purity markers using the following antibodies: anti-A2B5 (1:20; Miltenibiotec), anti-GLAST (1:20; Miltenibiotec), anti-CD44 (1:20; BD Pharmingen), anti-CXCR4 (1:20; Biolegend), anti-TRA-1-60 (1:50; Biolegend), anti-EPCAM (1:50; Biolegend), anti-SSEA4 (1:50; Biolegend), anti-GFAP (1:2000; Sigma), Nestin (1:500; BD Pharmingen) and anti-AQP-4 (1:2000; Abcam). The Flow Cytometer FACS Canto II (BD) was operated with FACSDIVA software (BD). At least 10,000 events were collected per sample.

### Glutamate uptake assay

Glutamate uptake capability of the cells was measured in 28-day differentiated hESC-derived astrocytes. Glutamic acid (0.5 mM; Sigma) in Hanks’ Balanced Salt Solution (Gibco) was added to 1 × 10^6^ cells/ml. After 0, 10, 30, 60 and 120 min, the solution was aspirated and kept at 4 °C until further testing. Human astrocytes derived from the spinal cord (from Thermo Scientific) served as positive control, while 0.5 mM glutamic acid kept at 37 °C for 120 min served as negative control. In addition, 0.5 mM glutamic acid kept at 4 °C for 120 min served as time 0 concentration control. The EnzyChrom™ Glutamate Assay Kit (BioAssay Systems) was used to measure the concentration of glutamate in the collected samples according to the manufacturer’s protocol and recommendations. The optical density was read at 565 nm using the iMark Microplate reader (Bio Rad). Dihydrokainic acid (DHK, 500 μM; Sigma) or 1 μM WAY-213,613 (Sigma) were used as inhibitors of GLT-1.

### Secretome analysis

In order to promote astrocyte differentiation, APCs were deprived from growth factors (bFGF and EGF) and vitamin C was added for 7 days and 28 days. Conditioned media were collected after 48 h from each experimental well. The number of cells for each well was counted (at least two replicas per each cell type) and secretome analysis was performed by multiplex ELISA using the human quantibody kiloplex Array (Raybiotech). The values obtained in secretome analysis were normalized to 1 × 10^6^ cells/ml.

### Transplantation of hES-AS in the hSOD1^G93A^ animal model

Transgenic hSOD1^G93A^ mice aged 8–9 weeks of mixed gender (B6SJL-Tg(SOD1*G93A)1Gur/J) were purchased from The Jackson Laboratory (Bar Harbor, ME, USA; https://www.jax.org/). Transgenic hSOD1^G93A^ rats aged 5–6 weeks of mixed gender (NTac:SD-Tg(SOD1^G93A^)L26H) were purchased from Taconic Biosciences Inc. (Hudson, NY, USA; http://www.taconic.com). All animal care and surgical procedures described here were carried out according to protocols approved by the Israeli National Committee for Animal Care. The animals were kept in a certified animal facility in IVC cages with a light cycle of 12 h and at temperature of 22 ± 2 °C. Rodent diet and drinking water were provided ad libitum.

#### Intrathecal injection through the cisterna magna

Mice were anesthetized with an i.p. injection of ketamine/xylazine (K4138; Sigma) and then mounted on a stereotaxic frame. The head was then bent, resulting in nape distention. A midline skin incision was made at the nape area to expose the sagittal suture of the cranium and midline of the nape. Under a dissection microscope, the subcutaneous tissue and muscles were separated by blunt dissection with forceps to expose the cleft between the occipital bone and the atlas vertebra. The muscles were held apart to expose the dura mater which was carefully penetrated using a 29G-gauge 45° beveled needle (Hamilton, Reno, NV, USA) connected to a 10-μl Hamilton syringe preloaded with 10 μl of cell suspension or vehicle (DMEM/F12 medium). Then 2 × 10^6^ hES-AS (APCs differentiated for 7 days) were injected once on day 67 ± 2 (CellsX1 group, *n* = 14 mice) or twice on day 67 ± 2 and on day 97 ± 2 at interval of 30 days (CellsX2 group, *n* = 13), or injected with DMEM F12 (Sham group, *n* = 10) into the CSF through the CM. The syringe was held in position for 3 min before being gradually pulled away to avoid liquid outflow along the needle tract. The skin cut was secured with stainless steel surgical clips and wiped with 70% ethanol.

#### Injection of the cells by lumbar puncture

The rats were anesthetized with ketamine/xylazine. The lumbar region was shaved, sterilized with iodine and the intervertebral spaces widened by placing the animal on a 15-ml conical plastic tube. The injections were performed by inserting a 29-gauge 45° beveled needle (Hamilton) connected to a 10-μl Hamilton syringe into the tissues between the dorsal aspects of L5 and L6. Correct subarachnoid positioning of the tip of the needle was verified by a tail flick test. A volume of 10 μl containing 3 × 10^6^ APCs was injected twice on day 50 ± 2 and on day 70 ± 2 (*n* = 7), or vehicle (DMEM/12 medium, *n* = 7) was injected. The syringe was held in position for 30 s before being progressively pulled away.

#### Immunosuppression

Immunosuppression was used only in the transplantation experiment in SOD1^G93A^ mice. In this experiment, Cyclosporin A was given daily by intraperitoneal injection, at a dose of 10 mg/kg, starting 3 days prior to the treatment and lasting all throughout the duration of the experiment. CellCept was administered orally twice a day at a dose of 15 mg/kg (total daily dose was 30 mg/kg). Dosing started 3 days prior to the treatment and lasted for a total of 10 consecutive days. Cohort 3, which was given the treatment twice, started receiving CellCept 3 days prior to each treatment injection for 10 consecutive days.

#### Measurements

Measurement of body weight and all motor tests took place 7–10 days prior to cell transplantation and routinely afterward. Motor function was tested using an acceleration Rotarod device (Rotarod 7650; Ugo Basile, Comerio, Italy) for the duration of 180 s. The time it took each mouse to fall from the rod was recorded. Animals were trained for 1 week prior to conducting the test. Forelimb muscle grip strength was determined using a Grip Strength Meter 47,200 (UGO Basile). Grip strength testing was performed by allowing the animals to grasp a thin bar attached to the force gauge. This is done by pulling the animal away from the gauge until the mice forelimbs released the bar. The procedure provides a value of the force of maximal grip strength. The force measurements were recorded in three separate trials, and the averages were used in the statistical analysis. Neurological scoring was done according to neurological score on a scale from 0 to 5 [99].

### Statistical analysis

Kaplan–Meier analysis of the SOD1^G93A^ mice and rats was conducted using the statistical software Sigmastat (SAS Software) to analyze survival, disease onset and duration data. Weight, time to fall from the Rotarod, neurological score and grip strength results were analyzed via repeated-measures ANOVA. All data are presented as mean ± SEM, and significance level was set at *p* ≤ 0.05. Statistical analysis was performed by MediStat Ltd, Israel.

#### Transplantation of hES-AS in NSG mice

The mouse was mounted on a stereotaxic frame. A midline skin incision was made at the nape area to expose the sagittal suture of the cranium and midline of the nape. The head was then bent, resulting in nape distention. Under a dissection microscope, the subcutaneous tissue and muscles were separated by blunt dissection with forceps to expose the cleft between the occipital bone and the atlas vertebra. The muscles were held apart to expose the dura mater which was penetrated using a 29G needle connected to a Hamilton syringe, preloaded with 10 μl of 0.4 × 10^6^ hES-AS. The cells were injected within 30 s into the CSF space. The needle was held for about 30 s after injection and then withdrawn. The skin cut was secured with stainless steel surgical clips and wiped with polydine solution.

## Additional files

Additional file 1: **Figure S1.** hES-AS produce and secrete neurotrophic factors. Conditioned media of 24 h from cultures of hES-AS differentiated for 28 days as well as cell extracts used to measure level of neurotrophic factors GDNF, BDNF, VEGF and IGF-1. For each factor, bars show cell content, amount secreted and negative control (medium only), expressed in pg/10^6^ cells (triplicates ± SD) (PDF 91 kb)

Additional file 2: **Table S1.** Secretome analysis of hES-AS, differentiated for 7 days or 28 days. The 220 most secreted factors by the 7-day differentiated hES-AS sorted by mean ng/ml/10^6^ cells ± SD (PDF 140 kb)

Additional file 3: **Figure S2.** Effect of hES-AS transplantation on disease onset, progression and survival in hSOD1^G93A^ mice. hES-AS, differentiated for 7 days, transplanted intrathecally through CM of hSOD1^G93A^ mice. A Three experimental groups tested, single injection of 2 × 10^6^ hES-AS on day 67 of life (Cellsx1), two injections of 2 × 10^6^ hES-AS each on days 67 and 97 (Cellsx2) and once sham-injected mice (vehicle). Kaplan–Meir plot of disease onset (measured by 3% body weight loss from maximal weight) showing more delay in twice-injected group. B Kaplan–Meier survival curves with similar trends. C Body weight maintained longer in hES-AS-treated mice. Note that a few days after second injection, day 97, weight loss occurred related to injection. D Neurological score. E Significant improvement in motor performance (Rotarod test) for hSOD1 mice transplanted twice with hES-AS. C, D Values are mean ± SEM (PDF 262 kb)

Additional file 4: **Table S2.** Percent of cell presence and percent of frequency scores greater than, or equal to ‘2’ (one to three foci of 10-20 cells per foci) for each follow up time (4, 17 and 39 weeks after hES-AS transplantation). Supplementary materials and methods. (ZIP 150 kb)

## Abbreviations

ALS: Amyotrophic lateral sclerosis
APC: Astrocyte progenitor cell
AQP-4: Aquaporin-4
CM: Cisterna magna
CNS: Central nervous system
CSF: Cerebrospinal fluid
G93A mutation: Glycine 93 changed to alanine
GFAP: Glial Fibrillary Acidic Protein
GLP: Good laboratory practice
GLT-1: Glutamate transporter 1
GMP: Good manufacturing practice
hES-AS: Human embryonic stem cell-derived astrocytes (differentiated from APCs for 7 days)
hESC: Human embryonic stem cell
hSOD1: Human superoxide dismutase 1
LOD: Limit of detection
LP: Lumbar puncture
MN: Motor neuron
NTF: Neurotrophic factor
SOD1: Superoxide dismutase 1
TIMP: Tissue inhibitor of metalloproteinase
VEGF: Vascular endothelial growth factor

## References

[1] Rowland LP, Shneider NA (2001). Amyotrophic lateral sclerosis. N Engl J Med.

[2] Rosen DR (1993). Mutations in Cu/Zn superoxide dismutase gene are associated with familial amyotrophic lateral sclerosis. Nature.

[3] Lagier-Tourenne C, Cleveland DW (2009). Rethinking ALS: the FUS about TDP-43. Cell.

[4] Renton AE, Chio A, Traynor BJ (2014). State of play in amyotrophic lateral sclerosis genetics. Nat Neurosci.

[5] Haidet-Phillips AM (2011). Astrocytes from familial and sporadic ALS patients are toxic to motor neurons. Nat Biotechnol.

[6] Meyer K (2014). Direct conversion of patient fibroblasts demonstrates non-cell autonomous toxicity of astrocytes to motor neurons in familial and sporadic ALS. Proc Natl Acad Sci U S A.

[7] Di Giorgio FP, Carrasco MA, Siao MC, Maniatis T, Eggan K (2007). Non-cell autonomous effect of glia on motor neurons in an embryonic stem cell-based ALS model. Nat Neurosci.

[8] Nagai M (2007). Astrocytes expressing ALS-linked mutated SOD1 release factors selectively toxic to motor neurons. Nat Neurosci.

[9] Di Giorgio FP, Boulting GL, Bobrowicz S, Eggan KC (2008). Human embryonic stem cell-derived motor neurons are sensitive to the toxic effect of glial cells carrying an ALS-causing mutation. Cell Stem Cell.

[10] Marchetto MC (2008). Non-cell-autonomous effect of human SOD1 G37R astrocytes on motor neurons derived from human embryonic stem cells. Cell Stem Cell.

[11] Papadeas ST, Kraig SE, O'Banion C, Lepore AC, Maragakis NJ (2011). Astrocytes carrying the superoxide dismutase 1 (SOD1G93A) mutation induce wild-type motor neuron degeneration in vivo. Proc Natl Acad Sci U S A.

[12] Yamanaka K (2008). Mutant SOD1 in cell types other than motor neurons and oligodendrocytes accelerates onset of disease in ALS mice. Proc Natl Acad Sci U S A.

[13] Tong J (2013). Expression of ALS-linked TDP-43 mutant in astrocytes causes non-cell-autonomous motor neuron death in rats. EMBO J.

[14] Rossi S, Cozzolino M, Carri MT (2016). Old versus new mechanisms in the pathogenesis of ALS. Brain Pathol.

[15] Foran E, Trotti D (2009). Glutamate transporters and the excitotoxic path to motor neuron degeneration in amyotrophic lateral sclerosis. Antioxid Redox Signal.

[16] Rothstein JD, Van Kammen M, Levey AI, Martin LJ, Kuncl RW (1995). Selective loss of glial glutamate transporter GLT-1 in amyotrophic lateral sclerosis. Ann Neurol.

[17] Lin CL (1998). Aberrant RNA processing in a neurodegenerative disease: the cause for absent EAAT2, a glutamate transporter, in amyotrophic lateral sclerosis. Neuron.

[18] Howland DS (2002). Focal loss of the glutamate transporter EAAT2 in a transgenic rat model of SOD1 mutant-mediated amyotrophic lateral sclerosis (ALS). Proc Natl Acad Sci U S A.

[19] Raoul C (2002). Motoneuron death triggered by a specific pathway downstream of Fas. potentiation by ALS-linked SOD1 mutations. Neuron.

[20] Barbeito LH (2004). A role for astrocytes in motor neuron loss in amyotrophic lateral sclerosis. Brain Res Brain Res Rev.

[21] Endo F (2015). Astrocyte-derived TGF-β1 accelerates disease progression in ALS mice by interfering with the neuroprotective functions of microglia and T cells. Cell Rep.

[22] Tripathi P (2017). Reactive astrocytes promote ALS-like degeneration and intracellular protein aggregation in human motor neurons by disrupting autophagy through TGF-β1. Stem cell reports.

[23] Phatnani, H.P., et al. Intricate interplay between astrocytes and motor neurons in ALS. Proc Natl Acad Sci U S A 110, E756–EE765 (2013). 10.1073/pnas.1222361110 PMC3581928 23388633

[24] Brambilla L (2016). Disruption of the astrocytic TNFR1-GDNF axis accelerates motor neuron degeneration and disease progression in amyotrophic lateral sclerosis. Hum Mol Genet.

[25] Das MM, Svendsen CN (2015). Astrocytes show reduced support of motor neurons with aging that is accelerated in a rodent model of ALS. Neurobiol Aging.

[26] Van Den Bosch L (2004). Effects of vascular endothelial growth factor (VEGF) on motor neuron degeneration. Neurobiol Dis.

[27] Oosthuyse B (2001). Deletion of the hypoxia-response element in the vascular endothelial growth factor promoter causes motor neuron degeneration. Nat Genet.

[28] Bogaert E (2010). VEGF protects motor neurons against excitotoxicity by upregulation of GluR2. Neurobiol Aging.

[29] Gros-Louis F (2003). Absence of mutations in the hypoxia response element of VEGF in ALS. Muscle Nerve.

[30] Llado J, Tolosa L, Olmos G (2013). Cellular and molecular mechanisms involved in the neuroprotective effects of VEGF on motoneurons. Front Cell Neurosci.

[31] Mishra PS (2016). Astroglia acquires a toxic neuroinflammatory role in response to the cerebrospinal fluid from amyotrophic lateral sclerosis patients. J Neuroinflammation.

[32] Re DB (2014). Necroptosis drives motor neuron death in models of both sporadic and familial ALS. Neuron.

[33] Hitomi J (2008). Identification of a molecular signaling network that regulates a cellular necrotic cell death pathway. Cell.

[34] Wiedemann FR, Manfredi G, Mawrin C, Beal MF, Schon EA (2002). Mitochondrial DNA and respiratory chain function in spinal cords of ALS patients. J Neurochem.

[35] Hirano A (1984). Fine structural study of neurofibrillary changes in a family with amyotrophic lateral sclerosis. J Neuropathol Exp Neurol.

[36] Sasaki S, Horie Y, Iwata M (2007). Mitochondrial alterations in dorsal root ganglion cells in sporadic amyotrophic lateral sclerosis. Acta Neuropathol.

[37] Tan W (2013). Small peptides against the mutant SOD1/Bcl-2 toxic mitochondrial complex restore mitochondrial function and cell viability in mutant SOD1-mediated ALS. The Journal of neuroscience : the official journal of the Society for Neuroscience.

[38] Nicaise C, Mitrecic D, Falnikar A, Lepore AC (2015). Transplantation of stem cell-derived astrocytes for the treatment of amyotrophic lateral sclerosis and spinal cord injury. World J Stem Cells.

[39] Tannenbaum SE (2012). Derivation of xeno-free and GMP-grade human embryonic stem cells--platforms for future clinical applications. PLoS One.

[40] Prathalingam N (2012). Production and validation of a good manufacturing practice grade human fibroblast line for supporting human embryonic stem cell derivation and culture. Stem Cell Res Ther.

[41] Izrael M (2007). Human oligodendrocytes derived from embryonic stem cells: effect of noggin on phenotypic differentiation in vitro and on myelination in vivo. Mol Cell Neurosci.

[42] Scholze AR, Foo LC, Mulinyawe S, Barres BA (2014). BMP signaling in astrocytes downregulates EGFR to modulate survival and maturation. PLoS One.

[43] Alfei L (1999). Hyaluronate receptor CD44 is expressed by astrocytes in the adult chicken and in astrocyte cell precursors in early development of the chick spinal cord. Eur J Histochem.

[44] Piao C (2015). Thrombin decreases expression of the glutamate transporter GLAST and inhibits glutamate uptake in primary cortical astrocytes via the Rho kinase pathway. Exp Neurol.

[45] Baba H (1997). GFAP gene expression during development of astrocyte. Dev Neurosci.

[46] Hubbard JA, Hsu MS, Seldin MM, Binder DK. Expression of the astrocyte water channel Aquaporin-4 in the mouse brain. ASN Neuro. 2015;7(5). 10.1177/1759091415605486 PMC4623559 26489685

[47] Gropp M (2012). Standardization of the teratoma assay for analysis of pluripotency of human ES cells and biosafety of their differentiated progeny. PLoS One.

[48] Kawahara K (2002). Selective blockade of astrocytic glutamate transporter GLT-1 with dihydrokainate prevents neuronal death during ouabain treatment of astrocyte/neuron cocultures. Glia.

[49] Straten G, Eschweiler GW, Maetzler W, Laske C, Leyhe T (2009). Glial cell-line derived neurotrophic factor (GDNF) concentrations in cerebrospinal fluid and serum of patients with early Alzheimer's disease and normal controls. J Alzheimers Dis.

[50] Li G (2009). Cerebrospinal fluid concentration of brain-derived neurotrophic factor and cognitive function in non-demented subjects. PLoS One.

[51] Gurney ME (1994). Motor neuron degeneration in mice that express a human Cu, Zn superoxide dismutase mutation. Science.

[52] Writing G, Edaravone ALSSG (2017). Safety and efficacy of edaravone in well defined patients with amyotrophic lateral sclerosis: a randomised, double-blind, placebo-controlled trial. The Lancet Neurology.

[53] Bensimon G, Lacomblez L, Meininger V (1994). A controlled trial of riluzole in amyotrophic lateral sclerosis. ALS/Riluzole Study Group. N Engl J Med.

[54] Lacomblez L, Bensimon G, Leigh PN, Guillet P, Meininger V (1996). Dose-ranging study of riluzole in amyotrophic lateral sclerosis. Amyotrophic Lateral Sclerosis/Riluzole Study Group II. Lancet.

[55] Miller RG, Mitchell JD, Moore DH. Riluzole for amyotrophic lateral sclerosis (ALS)/motor neuron disease (MND). The Cochrane database of systematic reviews. 2012;14(3):CD001447.

[56] Petrov D, Mansfield C, Moussy A, Hermine O (2017). ALS clinical trials review: 20 years of failure. are we any closer to registering a new treatment?. Front Aging Neurosci.

[57] Izrael M, Slutsky S, Itskovitz-Eldor J. & Revel M. Astrocytes in Pathogenesis of Neurodegenerative Diseases and Potential: Translation into Clinic. InTechOpen: Astrocyte—Physiology and Pathology, ISBN 978–953–51-5760-1 (Mar 2018).

[58] Lee J (2016). Astrocytes and microglia as non-cell autonomous players in the pathogenesis of ALS. Experimental neurobiology.

[59] Do-Ha D, Buskila Y, Ooi L. Impairments in motor neurons, interneurons and astrocytes contribute to hyperexcitability in ALS: underlying mechanisms and paths to therapy. Mol Neurobiol. 2017;55(2):1410–18. 10.1007/s12035-017-0392-y 28160214

[60] Storkebaum E, Lambrechts D, Carmeliet P (2004). VEGF: once regarded as a specific angiogenic factor, now implicated in neuroprotection. BioEssays.

[61] Krakora D, et al. Synergistic effects of GDNF and VEGF on lifespan and disease progression in a familial ALS rat model. Molecular therapy : the journal of the American Society of Gene Therapy. 2013;21(8):1602–10. 10.1038/mt.2013.108 PMC3734670 23712039

[62] Ramamohan PY (2007). Cerebrospinal fluid from amyotrophic lateral sclerosis patients causes fragmentation of the Golgi apparatus in the neonatal rat spinal cord. Amyotrophic lateral sclerosis : official publication of the World Federation of Neurology Research Group on Motor Neuron Diseases.

[63] Deepa P (2011). Down regulation of trophic factors in neonatal rat spinal cord after administration of cerebrospinal fluid from sporadic amyotrophic lateral sclerosis patients. J Neural Transm.

[64] Shruthi S (2017). Brain-derived neurotrophic factor facilitates functional recovery from ALS-cerebral spinal fluid-induced neurodegenerative changes in the NSC-34 motor neuron cell line. Neurodegener Dis.

[65] Rosito M, Deflorio C, Limatola C, Trettel F (2012). CXCL16 orchestrates adenosine A3 receptor and MCP-1/CCL2 activity to protect neurons from excitotoxic cell death in the CNS. The Journal of neuroscience : the official journal of the Society for Neuroscience.

[66] Christopherson KS (2005). Thrombospondins are astrocyte-secreted proteins that promote CNS synaptogenesis. Cell.

[67] Tyzack GE (2014). Astrocyte response to motor neuron injury promotes structural synaptic plasticity via STAT3-regulated TSP-1 expression. Nat Commun.

[68] Hafner A (2013). Neuroprotective role of gamma-enolase in microglia in a mouse model of Alzheimer's disease is regulated by cathepsin X. Aging Cell.

[69] Morisaki Y (2016). Selective expression of Osteopontin in ALS-resistant motor neurons is a critical determinant of late phase neurodegeneration mediated by matrix metalloproteinase-9. Sci Rep.

[70] Kaplan A (2014). Neuronal matrix metalloproteinase-9 is a determinant of selective neurodegeneration. Neuron.

[71] Wright MC (2014). Novel roles for osteopontin and clusterin in peripheral motor and sensory axon regeneration. The Journal of neuroscience : the official journal of the Society for Neuroscience.

[72] Meller R (2005). Neuroprotection by osteopontin in stroke. Journal of cerebral blood flow and metabolism : official journal of the International Society of Cerebral Blood Flow and Metabolism.

[73] Gardner J, Ghorpade A (2003). Tissue inhibitor of metalloproteinase (TIMP)-1: the TIMPed balance of matrix metalloproteinases in the central nervous system. J Neurosci Res.

[74] Israelson A (2015). Macrophage migration inhibitory factor as a chaperone inhibiting accumulation of misfolded SOD1. Neuron.

[75] Cordero-Llana O (2011). Clusterin secreted by astrocytes enhances neuronal differentiation from human neural precursor cells. Cell Death Differ.

[76] Winkler C, Yao S (2014). The midkine family of growth factors: diverse roles in nervous system formation and maintenance. Br J Pharmacol.

[77] Philips T, Rothstein JD (2015). Rodent models of amyotrophic lateral sclerosis. Current protocols in pharmacology.

[78] Collins MA, An J, Hood BL, Conrads TP, Bowser RP (2015). Label-free LC-MS/MS proteomic analysis of cerebrospinal fluid identifies protein/pathway alterations and candidate biomarkers for amyotrophic lateral sclerosis. J Proteome Res.

[79] Tarasiuk J, Kulakowska A, Drozdowski W, Kornhuber J, Lewczuk P (2012). CSF markers in amyotrophic lateral sclerosis. J Neural Transm.

[80] Gao L, Zhou S, Cai H, Gong Z, Zang D. VEGF levels in CSF and serum in mild ALS patients. J Neurol Sci. 2014;346(1-2):216–20. 10.1016/j.jns.2014.08.031 25204587

[81] Varghese AM (2013). Chitotriosidase—a putative biomarker for sporadic amyotrophic lateral sclerosis. Clin Proteomics.

[82] Przyborski SA (2005). Differentiation of human embryonic stem cells after transplantation in immune-deficient mice. Stem Cells.

[83] Andrews PW (2005). Embryonic stem (ES) cells and embryonal carcinoma (EC) cells: opposite sides of the same coin. Biochem Soc Trans.

[84] Lee AS (2009). Effects of cell number on teratoma formation by human embryonic stem cells. Cell Cycle.

[85] Prokhorova TA (2009). Teratoma formation by human embryonic stem cells is site dependent and enhanced by the presence of Matrigel. Stem Cells Dev.

[86] Ramot Y (2017). Compact MRI for the detection of teratoma development following intrathecal human embryonic stem cell injection in NOD-SCID mice. Neurotoxicology.

[87] Priest CA, Manley NC, Denham J, Wirth ED 3rd, Lebkowski JS. Preclinical safety of human embryonic stem cell-derived oligodendrocyte progenitors supporting clinical trials in spinal cord injury. 2015;10(8):939–58. 10.2217/rme.15.57 26345388

[88] Staff NP (2016). Safety of intrathecal autologous adipose-derived mesenchymal stromal cells in patients with ALS. Neurology.

[89] Oh KW (2015). Phase I trial of repeated intrathecal autologous bone marrow-derived mesenchymal stromal cells in amyotrophic lateral sclerosis. Stem Cells Transl Med.

[90] Tian C (2013). Autologous bone marrow mesenchymal stem cell therapy in the subacute stage of traumatic brain injury by lumbar puncture. Experimental and clinical transplantation : official journal of the Middle East Society for Organ Transplantation.

[91] Wang S (2013). Umbilical cord mesenchymal stem cell transplantation significantly improves neurological function in patients with sequelae of traumatic brain injury. Brain Res.

[92] Roybon L (2013). Human stem cell-derived spinal cord astrocytes with defined mature or reactive phenotypes. Cell Rep.

[93] Gupta K, Chandran S, Hardingham GE (2013). Human stem cell-derived astrocytes and their application to studying Nrf2-mediated neuroprotective pathways and therapeutics in neurodegeneration. Br J Clin Pharmacol.

[94] Petrou P (2016). Safety and clinical effects of mesenchymal stem cells secreting neurotrophic factor transplantation in patients with amyotrophic lateral sclerosis: results of phase 1/2 and 2a clinical trials. JAMA neurology.

[95] Glass JD (2012). Lumbar intraspinal injection of neural stem cells in patients with amyotrophic lateral sclerosis: results of a phase I trial in 12 patients. Stem Cells.

[96] Mazzini L (2015). Human neural stem cell transplantation in ALS: initial results from a phase I trial. J Transl Med.

[97] Kondo T (2014). Focal transplantation of human iPSC-derived glial-rich neural progenitors improves lifespan of ALS mice. Stem cell reports.

[98] Nizzardo M (2016). iPSC-derived LewisX+CXCR4+β1-integrin+ neural stem cells improve the amyotrophic lateral sclerosis phenotype by preserving motor neurons and muscle innervation in human and rodent models. Hum Mol Genet.

[99] Scott S (2008). Design, power, and interpretation of studies in the standard murine model of ALS. Amyotrophic lateral sclerosis : official publication of the World Federation of Neurology Research Group on Motor Neuron Diseases.

