# Supplementary figures and images for: Safety and efficacy of human embryonic stem cell-derived astrocytes following intrathecal transplantation in SOD1G93A and NSG animal models
Source: Stem Cell Res Ther. 2018 Jun 6;9:152. doi: 10.1186/s13287-018-0890-5 (PMC5989413; doi:10.1186/s13287-018-0890-5)

Neurotrophic factors synthesis and secretion

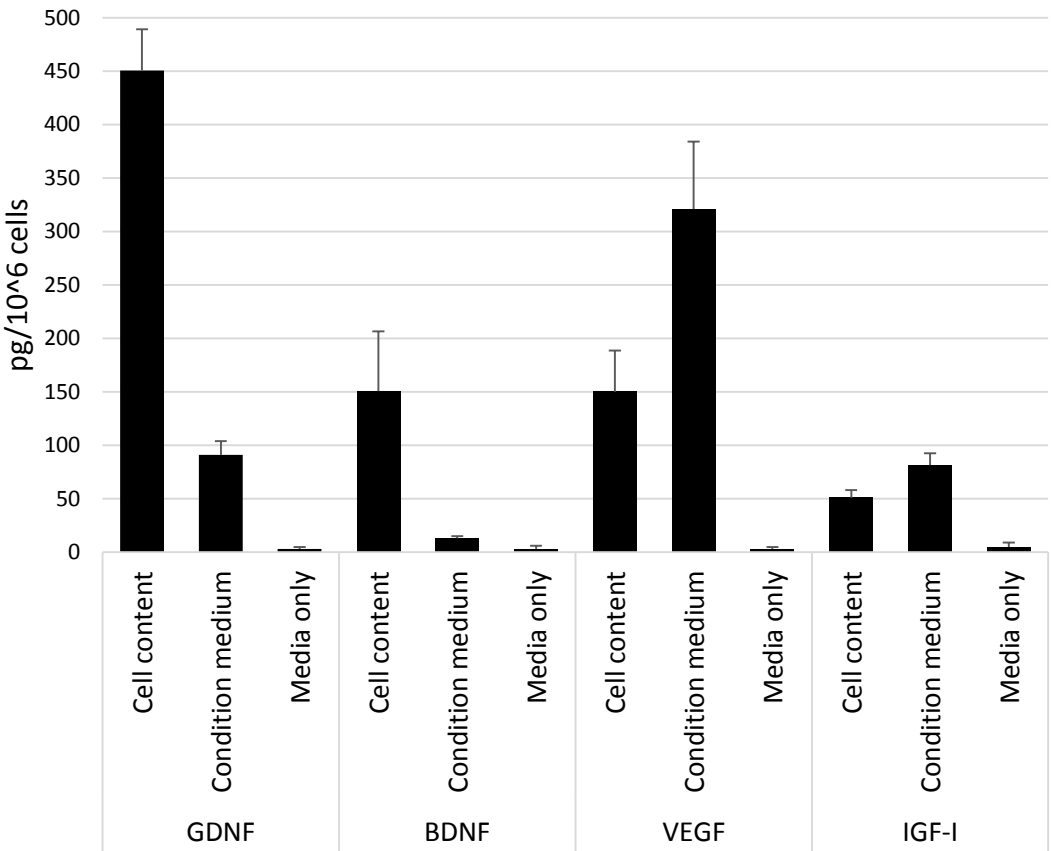

Supplement: Supplementary file 1 — Figure S1. hES-AS produce and secrete neurotrophic factors. Conditioned media of 24 h from cultures of hES-AS differentiated for 28 days as well as cell extracts used to measure level of neurotrophic factors GDNF, BDNF, VEGF and IGF-1. For each factor, bars show cell content, amount secreted and negative control (medium only), expressed in pg/106 cells (triplicates ± SD) (PDF 91 kb) [file 13287_2018_890_MOESM1_ESM.pdf]

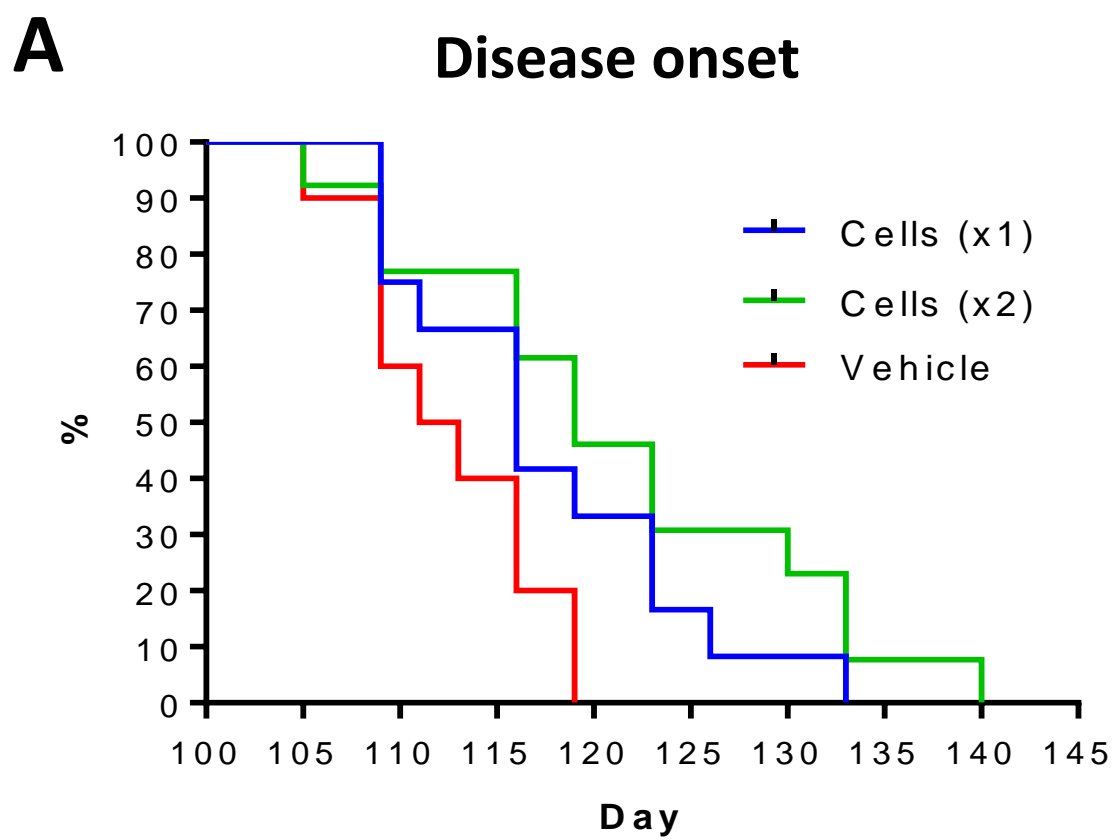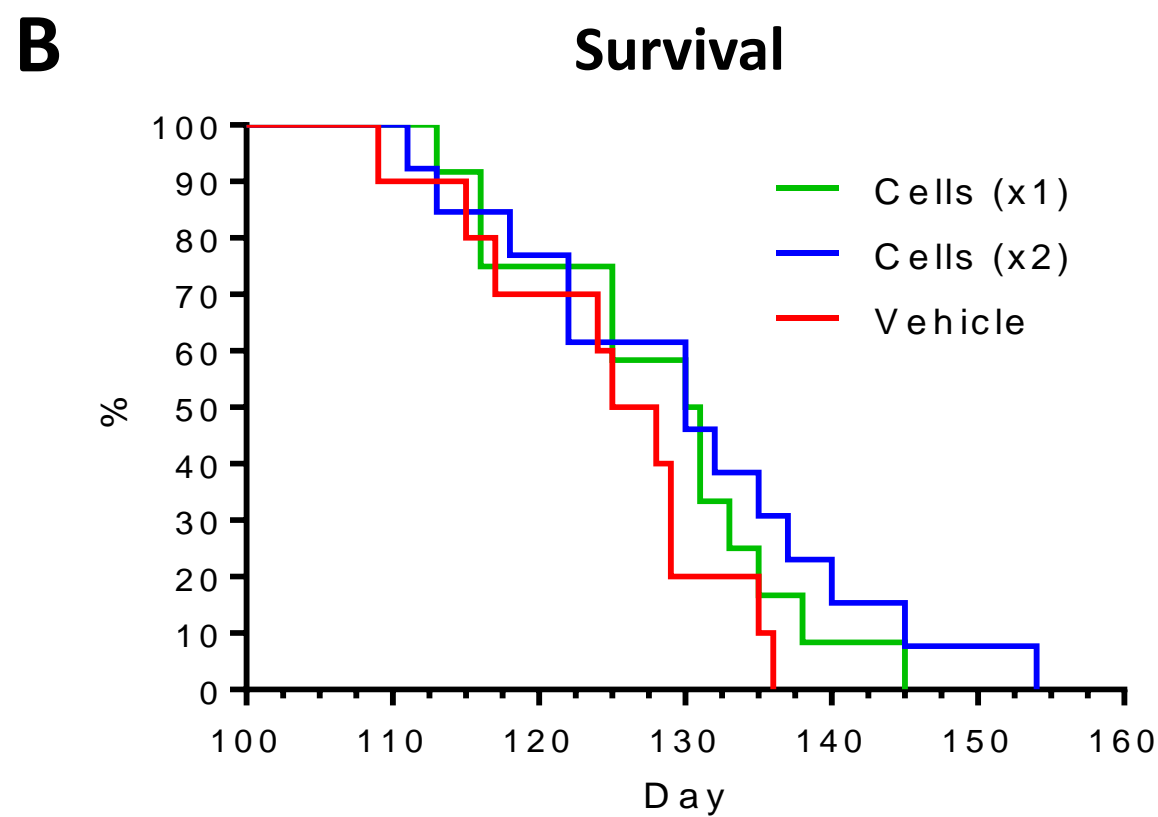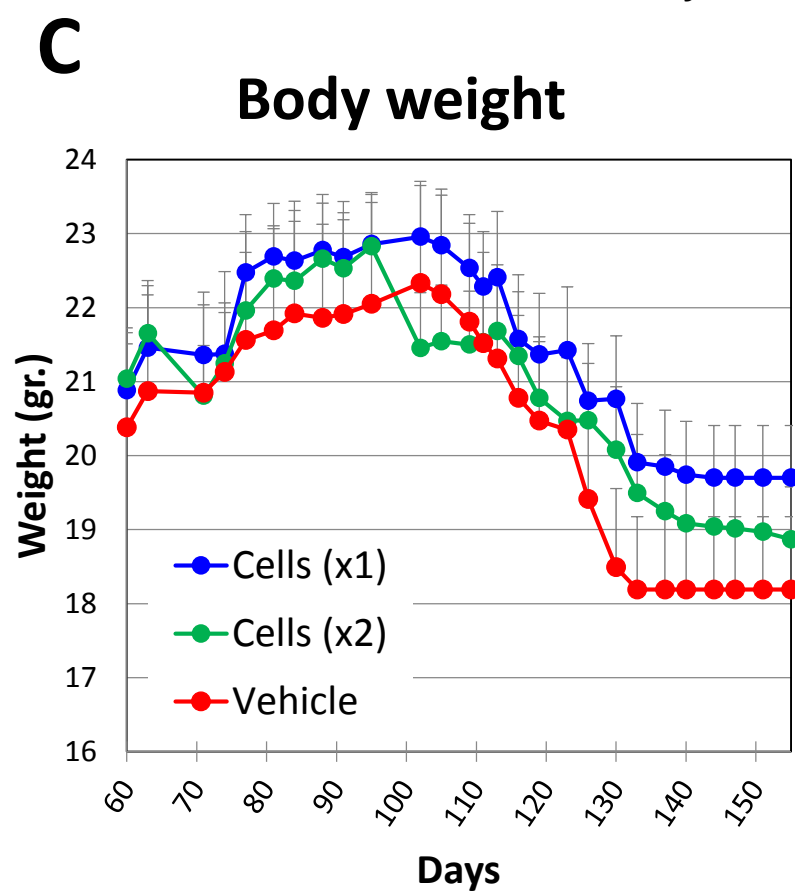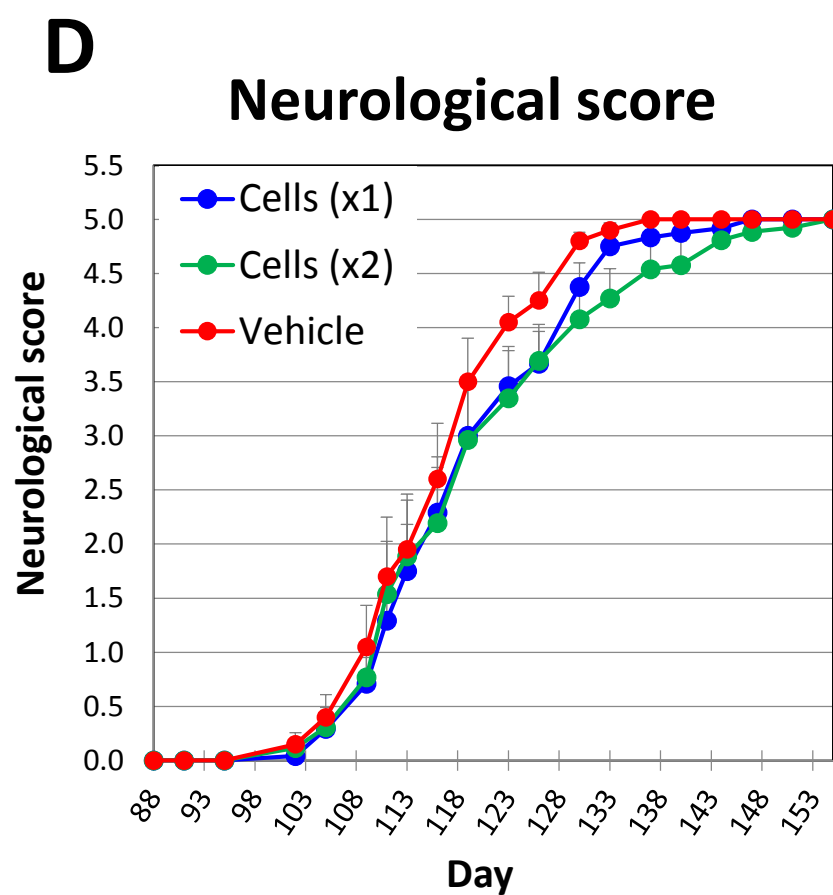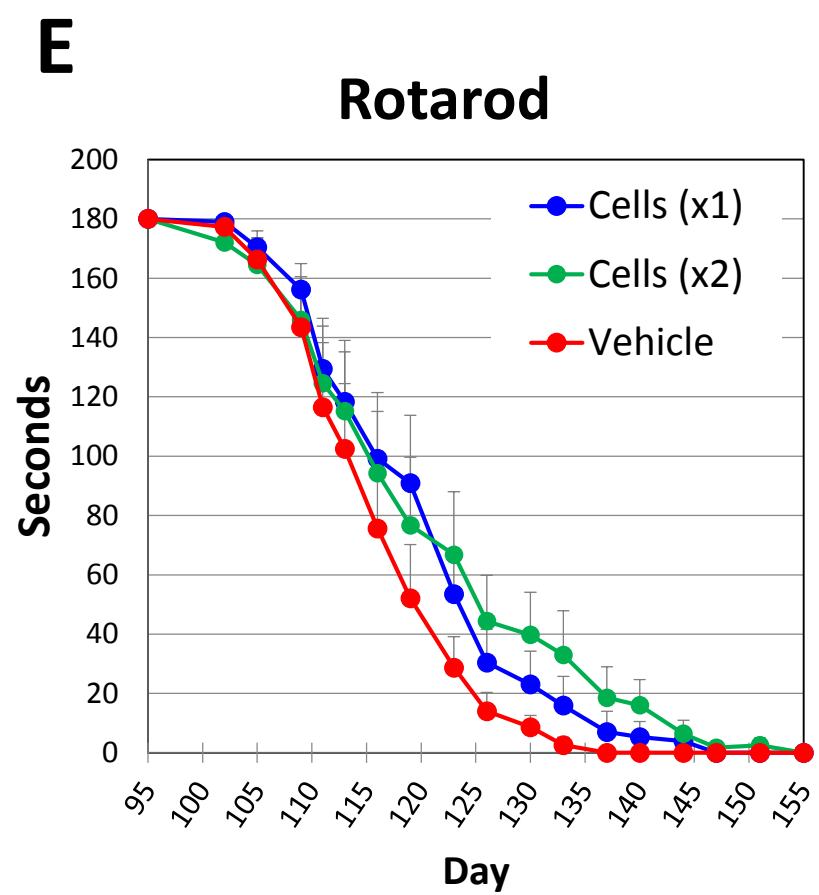

Supplement: Supplementary file 3 — Figure S2. Effect of hES-AS transplantation on disease onset, progression and survival in hSOD1G93A mice. hES-AS, differentiated for 7 days, transplanted intrathecally through CM of hSOD1G93A mice. A Three experimental groups tested, single injection of 2 × 10^6^ hES-AS on day 67 of life (Cellsx1), two injections of 2 × 10^6^ hES-AS each on days 67 and 97 (Cellsx2) and once sham-injected mice (vehicle). Kaplan–Meir plot of disease onset (measured by 3% body weight loss from maximal weight) showing more delay in twice-injected group. B Kaplan–Meier survival curves with similar trends. C Body weight maintained longer in hES-AS-treated mice. Note that a few days after second injection, day 97, weight loss occurred related to injection. D Neurological score. E Significant improvement in motor performance (Rotarod test) for hSOD1 mice transplanted twice with hES-AS. C, D Values are mean ± SEM (PDF 262 kb) [file 13287_2018_890_MOESM3_ESM.pdf]
