# Supplementary material for: Safety and efficacy of human embryonic stem cell-derived astrocytes following intrathecal transplantation in SOD1G93A and NSG animal models
Source: Stem Cell Res Ther. 2018 Jun 6;9:152. doi: 10.1186/s13287-018-0890-5 (PMC5989413; doi:10.1186/s13287-018-0890-5)
Supplement: Supplementary file 4 — Table S2. Percent of cell presence and percent of frequency scores greater than, or equal to ‘2’ (one to three foci of 10-20 cells per foci) for each follow up time (4, 17 and 39 weeks after hES-AS transplantation). Supplementary materials and methods. (ZIP 150 kb) [file 13287_2018_890_MOESM4_ESM.zip › 13287_2018_890_MOESM4_ESM.pdf]

| Treatment | Parameter | Brain \| Level 1 | Brain \| Level 2 | Brain \| Level 3 | Brain \| Level 4 | Brain \| Level 5 | Brain \| Level 6 | Brain \| Level 7 | Spinal Cord \| Cervical | Spinal Cord \| Thoracic | Spinal Cord \| Lumbar | Spinal Cord \| Sacral |
|---|---|---|---|---|---|---|---|---|---|---|---|---|
| hES-AS 4 weeks | Cell presence | 47% | 63% | 60% | 57% | 80% | 73% | 73% | 63% | 27% | 17% | 23% |
| | Frequency Score ≥ 2 | 21% | 16% | 6% | 0% | 17% | 5% | 5% | 5% | 0% | 0% | 0% |
| hES-AS 17 weeks | Cell presence | 50% | 90% | 93% | 80% | 97% | 97% | 87% | 67% | 30% | 20% | 13% |
| | Frequency Score ≥ 2 | 20% | 30% | 21% | 25% | 34% | 17% | 15% | 20% | 0% | 0% | 0% |
| hES-AS 39 weeks | Cell presence | 61% | 86% | 79% | 64% | 96% | 96% | 82% | 64% | 43% | 21% | 21% |
| | Frequency Score ≥ 2 | 47% | 63% | 45% | 11% | 59% | 33% | 57% | 28% | 17% | 0% | 0% |
