# Supplementary material for: Safety and efficacy of human embryonic stem cell-derived astrocytes following intrathecal transplantation in SOD1G93A and NSG animal models
Source: Stem Cell Res Ther. 2018 Jun 6;9:152. doi: 10.1186/s13287-018-0890-5 (PMC5989413; doi:10.1186/s13287-018-0890-5)
Supplement: Supplementary file 4 — Table S2. Percent of cell presence and percent of frequency scores greater than, or equal to ‘2’ (one to three foci of 10-20 cells per foci) for each follow up time (4, 17 and 39 weeks after hES-AS transplantation). Supplementary materials and methods. (ZIP 150 kb) [file 13287_2018_890_MOESM4_ESM.zip › 13287_2018_890_MOESM4_ESM.docx]

**Material and methods supplementary**

Neurotrophic factors synthesis and secretion assay

The contents of neurotrophic factors (GDNF, BDNF, VEGF and IGF-I) were measured in 1x10^6^ cells 28 day-differentiated astrocytes cells. We also measured the levels of neurotrophic factors secreted in 24 hours conditioned media, which was kept at -80°C until testing. Cells were harvested using TryplE , which was then inactivated with 4 volumes of 2 % KORS in DMEM/F12, and then the cells were resuspended in M-PER mammalian protein extraction reagent (all from Thermo Scientific) supplemented with 1:100 protein inhibitor cocktail (Sigma), and kept at -80°C until testing. Levels of the neurotrophic factors were measured using ELISA kit Quantikine (VEGF, human Free BDNF, and human IGF-I) and AbCam (human GDNF). All assays were performed according to manufacturer's protocol and recommendations. As negative control, we used media without cells. The optical density was read using Plate reader iMark Microplate reader (Bio Rad).

Neurite outgrowth test

*Rat cortical neurons isolation and culture*: Brains from embryonic day 18 embryos were isolated from Sprague Dawley (SD) rats, and preserved in Hibernate medium supplemented with 2% B27 and 0.5mM Glutamax (all from Invitrogen). After removing meninges, cortex was dissected and the clean tissue was enzymatically digested by adding three volumes of 0.25% trypsin. The enzymatic digestion was done for 10 minutes at 37ºC with repeated very gentle pipetting every 5 minutes. Trypsin was neutralized with one volume of DTI (Invitrogen) and two volumes of 2%BSA. DNase 1:500 was added, energetically re-suspended and left for additional 5 minutes at 37 ºC. The resulting suspension was centrifuged, the supernatant was discarded and the cell suspension was filtered through a 100µm mesh and centrifuged again. The resulting pellet was re-suspended in Neurobasal medium supplemented with 2%B27, 0.5mM Glutamax and 1% penicillin-streptomycin-amphotericine (Biological Industries, Israel). The resulting cell suspension was counted and the cell density was adjusted to 80,000 cells per well for seeding over poly-L-Lysine coated 96-well plates.

*Co-culture between rat cortical neurons and astrocytes*: hES-AS after 7 days of differentiation, were removed by trypsinization and added to the rat cortical neurons cultures (which had been cultured for 2 days before) at a density of ~8000 cells in each well of a 96-well plate (0.15 ml/well). The co-cultures, using 1-2x10^4^ hES-AS were done in the same Neurobasal medium used for the cortical neurons above. In parallel, neurons were cultured alone (negative control) or with 10ng/ml NT3 (PeproTech) (positive control). After 4 days, the cells were fixed and stained. Quantification of neurite outgrowth area was based on NF160 staining using 36 field pictures from each of 3 different wells in high content image screening system Arrayscan VTI (Thermo scientific, Cellomics) Statistical analysis was by Student's t-test.

Oxidative stress test

Mouse MNs were derived from E18 cortices and 20,000 neurons were seeded in a well of 96-well plate (0.15ml/well). The cultures were exposed to 150 µM H_2_O_2_ (Sigma) for 6 hours, or left untreated. During H_2_O_2_ treatment, neuron culture was supplemented with day 28 hES-AS conditioned medium (ACM) or with 20,000 hES-AS cells (day 28), or not supplemented. After 24 hours, the cultures were fixed and stained with anti-Tubulin β3, anti-Caspase-3a antibodies (Both from Abcam) and with DAPI. The percentage of apoptotic neurons (Caspase3a positive cells) were quantified using high content image screening microscope Arrayscan VTI, to quantify the number of caspase-3 positive cells versus total tubulin-β3 labeled neurons.

*Slide preparation for histopathological evaluation*

Organs including brain, spinal cord, spleen, kidney, testis/ovary, liver, heart, bone-marrow of the femur, lungs, and cervical lymph nodes were collected from all animals during the respective scheduled endpoint post hES-AS transplantation (4, 17 and 39 weeks). The organs were fixed in either 10% neutral buffered formalin or Davidson’s Solution. All tissues were trimmed, embedded in paraffin, sectioned at approximately 5 microns thickness, and then stained with Hematoxylin & Eosin (H&E). For CNS histopathology, the brain was sectioned in 7 representative levels according to Bolon et al, 2013. The spinal cord was transversely sectioned at the cervical, thoracic, lumbar and sacral areas, at 3 sections per area. All H&E slides underwent histopathological evaluation.

*Detection of hES-AS cells in distant mouse organs by qPCR*

Genomic DNA was extracted from the 10 organs of NSG mice including: whole blood, spleen, kidney, testis, ovary, liver, heart, femur (bone marrow), lung and cervical lymph node. Amplification of *Alu Y* sequence for detection of human cells in each mouse tissue was performed by Taqman PCR using 1 µg of total DNA per reaction and AluY-specific primers and probe. Each reaction contained Internal positive control (Applied Biosystems, 4308323) and a template-free negative control. A standard curve was constructed from human genomic DNA (Roche 11691112001) over a concentration range equivalent to 10,000 to 0.1 human cells, in a matrix of 1 µg gDNA from mouse liver. Limit of detection (LOD) was calculated as the lowest human DNA concentration giving >50 % amplification and was set at 0.1 cell. Limit of quantification (LOQ) was defined as the lowest human genomic DNA concentration providing 100% amplification by different analysts and was set at 1 cell.
